# A core outcome set for the wellbeing of healthcare professionals: an international Delphi consensus study

**DOI:** 10.1016/j.eclinm.2026.104145

**Published:** 2026-08-17

**Authors:** Tom Bazuin, Sarah Frederika Christina Mugge, Amber Charlotte Patricia Boskma, Jesús Lopez-Alcalde, Dave Anton Dongelmans, Lotty Hooft, Maarten Jurriaan van der Laan, The CROWN Steering Committee, Diptesh Aryal, Amanda Quintairos e Silva, Jorge Salluh, Lode Godderis, Hanan Abdul Rahim, Meredith Fendt-Newlin, Yohannes Molla Asemu, Jan Hoving, Rachel Rajadurai, Götz Wietasch, The CROWN Panellist Group, Alen Antony Joy, Alexis Germán Murillo Carrasco, Amanda Drost, Amanuel Sisay Endeshaw, Ana Castillo, Apurb Sharma, Augusto Ferreira Umpiérrez, Bashu Dev Parajuli, Belete Negese, Beltran Carrillo, Bikila Chemir, Cindy Frias, Cong Wang, Corey Feist, Demeke Fentie, Francisco Manuel Olmos Vega, Getahun Tulu Amante, Getaneh Mulu, Habtemaryam Tedla Mekonnen, Hailemariam Getachew Tesema, Henok Legesse, Ilse Kleibrink, Kalpana Kharbuja, Kamil Barański, Maharoff Ahameth Nafrin Ali, Makda Abate Belew, Maria Natividad Seisdedos Núñez, Marieke Dijkman, Marieke Timpener, Maritza Titus, Matteo Bruschettini, Meike Slettenhaar, Meseret Yitayew Tizazu, Mulugeta Abate Hailemariyam, Nadine van de Reep, Olivia Lavreysen, Pamela Seron Silva, Peter de Winter, Philip Ayieko, Prati Badan Dangol, Stefan Schröder, Rana Nashashibi, Rutger Spruit, Sanne Drost-Goossens, Senta Jorinde Raasveld, Shirish KC, Sithy Zulaiha Zuhair, Szymon Szemik, Vera Otermann, Yideg Abinew

**Affiliations:** a Department of Surgery, University Medical Centre Groningen, University of Groningen, Groningen, the Netherlands; b Julius Centre for Health Sciences and Primary Care, University Medical Centre Utrecht, Utrecht University, Utrecht, the Netherlands; c Department of Intensive Care Medicine, Amsterdam UMC Location University of Amsterdam, Amsterdam, the Netherlands; d Complementary and Integrative Digital Health, Institute of Primary Care – University of Zurich and University Hospital Zurich, Zurich, Switzerland; e Hospital Universitario Ramón y Cajal, IRYCIS, Faculty of Medicine, Universidad Francisco de Vitoria, Madrid, Spain; f Cochrane Netherlands, University Medical Centre Utrecht, Utrecht University, Utrecht, the Netherlands

**Keywords:** Core outcome set, Wellbeing, Healthcare professional, Delphi

## Abstract

**Background:**

Healthcare professionals’ (HCPs) wellbeing is under sustained pressure globally, with rising sickness absence, burnout, and attrition undermining patient safety, care quality, and system resilience. Outcome measurement is heterogeneous across studies, limiting comparability and synthesis. We aimed to develop an internationally applicable core outcome set (COS) for HCP wellbeing.

**Methods:**

We conducted an international, electronic, modified RAND/UCLA Delphi study. Panel members were recruited internationally between May 7, 2025, and July 26, 2025 through personal invitations, snowball sampling, and professional networks. Candidate constructs were collated from a published scoping review and an additional MEDLINE search on economic determinants, organised within the PERMA+4 framework, and provided with standardised definitions. The Delphi process consisted of three sequential survey rounds conducted electronically. Panellists (physicians, nurses, allied HCPs, and health-workforce researchers) rated construct importance on a 9-point scale. Consensus criteria were: include if ≥85% rated 7–9 and <15% rated 1–3; exclude if <50% rated 7–9; all others progressed. In round 1 (July–Sept 2025), the start set of constructs was rated. In round 2 (Sept–Oct 2025), panellists re-rated constructs with group feedback; in round 3 (Nov 2025–Jan 2026), panellists ranked included constructs and indicated priority constructs by level (individual, team, organisation, system). The final COS was determined by panel ranking and subsequently reviewed and endorsed by the Steering Committee.

**Findings:**

From 254 candidate constructs, we refined 137 unique constructs for Delphi rating. 57 (100% of invited) panellists completed round 1, 50 (88%) completed round 2, and 46 (81%) completed round 3. Panellists participated from 20 countries across six continents. Thirty constructs met inclusion criteria in round 1; two additional constructs were added from panellist suggestions. After round 2, 62 constructs remained for prioritisation. Ranking yielded a ten-item COS, led by work–life balance, job satisfaction, and quality of life; other prioritised outcomes included motivation, mental health, meaning at work, income satisfaction, work culture, perceived professional growth, and stress.

**Interpretation:**

This international consensus COS contributes to outcome homogeneity by defining a set of outcomes for HCP wellbeing across settings. Its use could enhance comparability, support evidence synthesis, and inform quality improvement, while allowing addition of locally meaningful outcomes. Consistent measurement will require subsequent selection of valid, feasible, and context-appropriate instruments for each outcome. Future work should thus select and validate feasible, context-appropriate measurement instruments for each outcome (a core outcome measurement set) and test their cross-cultural measurement properties.

**Funding:**

None.

**Research in context**

**Evidence before this study**

We considered evidence from a published scoping review that mapped how healthcare professionals’ wellbeing has been operationalised and measured in empirical studies. That review identified a large number of non-overlapping constructs and instruments, confirming substantial outcome heterogeneity that limits cross-study comparability and evidence synthesis. Because the purpose was evidence mapping, the available literature was largely descriptive, with no meaningful pooled effect estimates and limited value of formal risk-of-bias appraisal for the question of ‘what is being measured’. Because economic aspects of wellbeing were under-represented in the mapped construct landscape, we additionally searched MEDLINE for relevant papers published between database inception and Feb 4, 2025 and used similar wellbeing search terms as the scoping review, combined with economic terms (e.g., income, wages, salary, financial status, compensation). We screened titles/abstracts and full texts, including studies that reported outcomes related to both economic security and healthcare professional (HCP) wellbeing. We also considered prior consensus initiatives and systematic reviews of organisation-directed interventions, which underscored inconsistent outcome selection and limited cumulative learning.

**Added value of this study**

This study translates a comprehensive evidence-informed construct landscape into a feasible, internationally derived core outcome set (COS) spanning multiple professional roles and geo-economic regions. By combining structured definitions, prespecified consensus criteria, and a ranking step to resolve uniformly high ratings, we produced a practical ten-item COS that includes outcomes spanning lived wellbeing (e.g., work–life balance, quality of life), work experience (e.g., job satisfaction), organisational conditions as experienced by staff (e.g., perceived organisational support, leadership), and economic security (income satisfaction). We further characterised priorities by level of influence (individual, team, organisational), improving decision relevance.

**Implications of all the available evidence**

Adoption of this COS as a set in trials, implementation studies, and routine monitoring in daily practice could help to reduce outcome heterogeneity, improve comparability, and strengthen evidence synthesis. The COS should be complemented by context-specific modules and measurement of key determinants (e.g., workload, staffing, safety climate) to guide action and explain effects. More research is required. Next steps include selecting and validating (combined) instruments (a core outcome measurement set), testing cross-cultural measurement properties, and establishing feasible measurement schedules and accountability for acting on results.

## Introduction

The wellbeing of healthcare professionals (HCPs) is under sustained pressure worldwide, reflected in rising sickness absence, burnout, and workforce attrition.^1^, ^2^, ^3^, ^4^ Wellbeing in this context is inherently multidimensional, spanning how HCPs feel, function, and find meaning at work alongside their physical health and economic security, yet it is seldom defined explicitly.^5^ These trends jeopardise patient safety, quality of care, and the resilience of health systems.^6^, ^7^, ^8^ This challenge is expected to intensify in the coming years. The World Health Organization projects a global shortfall of 10 million health workers by 2030, pointing out that all health systems face persistent challenges in educating, employing, deploying, retaining, and supporting their workforce.^9^ At the organisational level, poor wellbeing among HCPs contributes to absenteeism, turnover, and substantial financial costs.^10^ At the individual level, it is associated with impaired work–life balance, somatic morbidity, and diminished quality of life.^11^ In the context of ageing populations and increasing multimorbidity, protecting HCP wellbeing is therefore both a moral imperative and a strategic necessity.

Measuring HCP wellbeing in a consistent way is essential for identifying needs, monitoring change over time, and evaluating the effects of interventions. Without such measurement, interventions risk being implemented without clear evidence of whether they address the right problems or improve outcomes. Despite broad recognition of the problem, measurement of HCP wellbeing remains heterogeneous.^12^, ^13^, ^14^ Studies variously operationalise mental health, physical health, job-related affect, engagement, and stress using non-overlapping outcomes and instruments.^15^ This variability in both outcomes and measurement instruments impedes comparison across interventions and settings, and constrains cumulative knowledge building. Conceptual frameworks can help to structure the field. The PERMA+4 framework offers a comprehensive lens for identifying all the relevant domains of wellbeing at work.^16^ It encompasses the following domains: Positive emotions, Engagement, Relationships, Meaning, Accomplishment, plus Physical health, Mindset, Work environment, and Economic security. Yet even with such guidance, there is no widely adopted, internationally applicable set of core outcomes for HCP wellbeing. As a result, research often privileges convenience in outcome selection, rather than consensus on what matters most to HCPs, organisations, and researchers.

Recent initiatives have begun to map the landscape, including work among doctors in the UK National Health Service (NHS).^17^ Although valuable, these efforts focus on selected subgroups within specific systems and therefore have limited generalisability across professions, countries, and income settings. What is needed is an agreed core outcome set (COS) that enables consistent, decision-relevant measurement across contexts, while accommodating additional, locally meaningful outcomes where appropriate.^18^ A COS reduces outcome heterogeneity by defining what should be measured, thereby enhancing comparability of studies and supporting evidence synthesis. It also provides a practical foundation for routine monitoring and quality improvement by identifying core aspects of wellbeing that should be followed over time. As such, a COS is an essential precursor to a core outcome measurement set, in which valid, feasible and context-appropriate instruments are selected for the COS.

By integrating evidence from the literature with international stakeholder consensus, this study aims to establish a COS for HCP wellbeing to enable consistent measurement, improve comparability across interventions and settings, and inform efforts to maintain and improve HCP wellbeing.

## Methods

### Study design

We conducted an international, electronic, modified RAND/UCLA Delphi study to develop a COS for the wellbeing among HCPs.^19^ The study was initiated and coordinated by a core research group (TB, SM, AB, DD, MvdL and LH) and supported by an international, multidisciplinary Steering Committee with expertise in wellbeing research and representation across professional roles and geo-economic regions (Appendix A in the Supplement).

The study was designed and reported in accordance with COS-STAD development standards (Supplementary table S1)^20^ and COS-STAR^21^ reporting guidelines (Supplementary Table S22), and followed recommendations from the COMET handbook for Delphi methodology. As this study uses a Delphi methodology, reporting of the Delphi process was additionally informed by the reporting standard for Conducting and REporting of Delphi Studies (CREDES).^22^ The study protocol was prospectively registered in the COMET database (https://www.comet-initiative.org/Studies/Details/3255) and on the Open Science Framework (OSF: https://osf.io/9c5pr).

### Ethics

Institutional approval was obtained from the Medical Ethical Committee of the University Medical Centre Groningen (reference:2024/669) (Appendix E). Participants provided informed consent prior to participation in the first Delphi round.

### Development of an outcome list

Potential constructs were identified through a prior scoping review^23^ and mapped to the PERMA+4 framework,^16^ which served as the conceptual framework for this study. This mapping showed that the domain of economic security was not adequately represented. Therefore, an additional targeted MEDLINE search was conducted for relevant papers published between database inception and Feb 4, 2025 to identify constructs related to economic determinants of wellbeing (Supplementary Table S2). The additional search was screened independently by two reviewers (TB, SM). Following consultation with the Steering Committee, all identified constructs were independently reviewed, deduplicated, and conceptually refined by three reviewers (TB, SM, AB), resulting in the Delphi start list (Supplementary Tables S3 and S4). Detailed methodology of construct identification and refinement is provided in the Supplementary material.

### Piloting

The Delphi survey was developed in Castor EDC^24^ and piloted by Steering Committee members to assess feasibility, clarity, and content validity. Feedback informed minor refinements to survey structure and instructions. The estimated completion time was 20–30 min.

### Recruitment of panel members

Panel members were recruited internationally between May 7, 2025, and July 26, 2025 through personal invitations, snowball sampling, and professional networks (including LinkedIn). Eligible participants were physicians, nurses, allied health professionals working in hospital or public health settings, and researchers in health workforce or wellbeing research. Participants registered via a secure online form and provided informed consent. Broad eligibility was intentional. HCP wellbeing is an interprofessional and cross-system concern, and we sought to include the range of stakeholders who would ultimately use or apply the COS: practising HCPs across professional roles and researchers who develop, evaluate, or apply wellbeing measures. To ensure that final prioritisation reflected practising HCP perspectives, researchers were excluded from the primary ranking analysis, and robustness to panel composition was examined through weighted and subgroup analyses by professional role and geo-economic region. Recruitment was monitored to promote representation across professional roles and geo-economic regions, including low-, middle- and high-income countries, defined by the World Bank.^25^ We aimed to include approximately 30 participants per stakeholder stratum (total target 90–120). This target was based on recommendations that 30 participants could provide stable consensus.^26^ Further details on stakeholder involvement and governance are provided in Appendix A with additional methodological details.

### Delphi procedure

The Delphi process consisted of three sequential survey rounds conducted electronically. Consensus criteria were predefined as follows: a construct was included if more than 85% of respondents rated it as critically important (scores of 7, 8, or 9 on a 9-point Likert scale) and fewer than 15% rated it as of low importance (scores of 1, 2, or 3 on a 9-point Likert scale). These thresholds were chosen based on thresholds commonly used in Delphi studies and were intended to balance inclusiveness in the early phase with sufficient discrimination for prioritisation.

In Round 1, panellists rated the importance of each construct on a 9-point Likert scale (1 = not important; 9 = critically important) or selected “I don’t know”. Participants could suggest additional constructs. To minimise response bias, construct order was randomised. Demographic information was collected at survey entry. Constructs meeting the consensus criteria were forwarded to Round 3. A construct was excluded if fewer than 50% of respondents rated it as critically important (scores of 7, 8, or 9). Constructs that did not meet either criterion were carried forward to Round 2. Suggested constructs were reviewed by the study team and added to Round 2 if they were mentioned by at least two panel members and were considered conceptually distinct from constructs already included in the list. In Round 2, constructs without consensus and eligible newly suggested constructs were re-rated. For each construct, participants were shown feedback from Round 1, including the percentage of panellists who had scored the construct as critically important (scores of 7, 8, or 9), the percentage who had scored it as of low importance (scores of 1, 2, or 3), and the median score, alongside their own previous rating. Only constructs that met the predefined criteria for inclusion were taken forward to Round 3. The Round 3 ranking pool therefore comprised all constructs that met the inclusion criterion in Round 1 or Round 2.

In Round 3, panellists ranked the remaining constructs to identify ten outcomes for the final COS, as prespecified to enhance feasibility and uptake.^27^ Participants also indicated the contextual level (e.g., individual, team, organisational) at which constructs were considered most relevant.

**Fig. 1:**
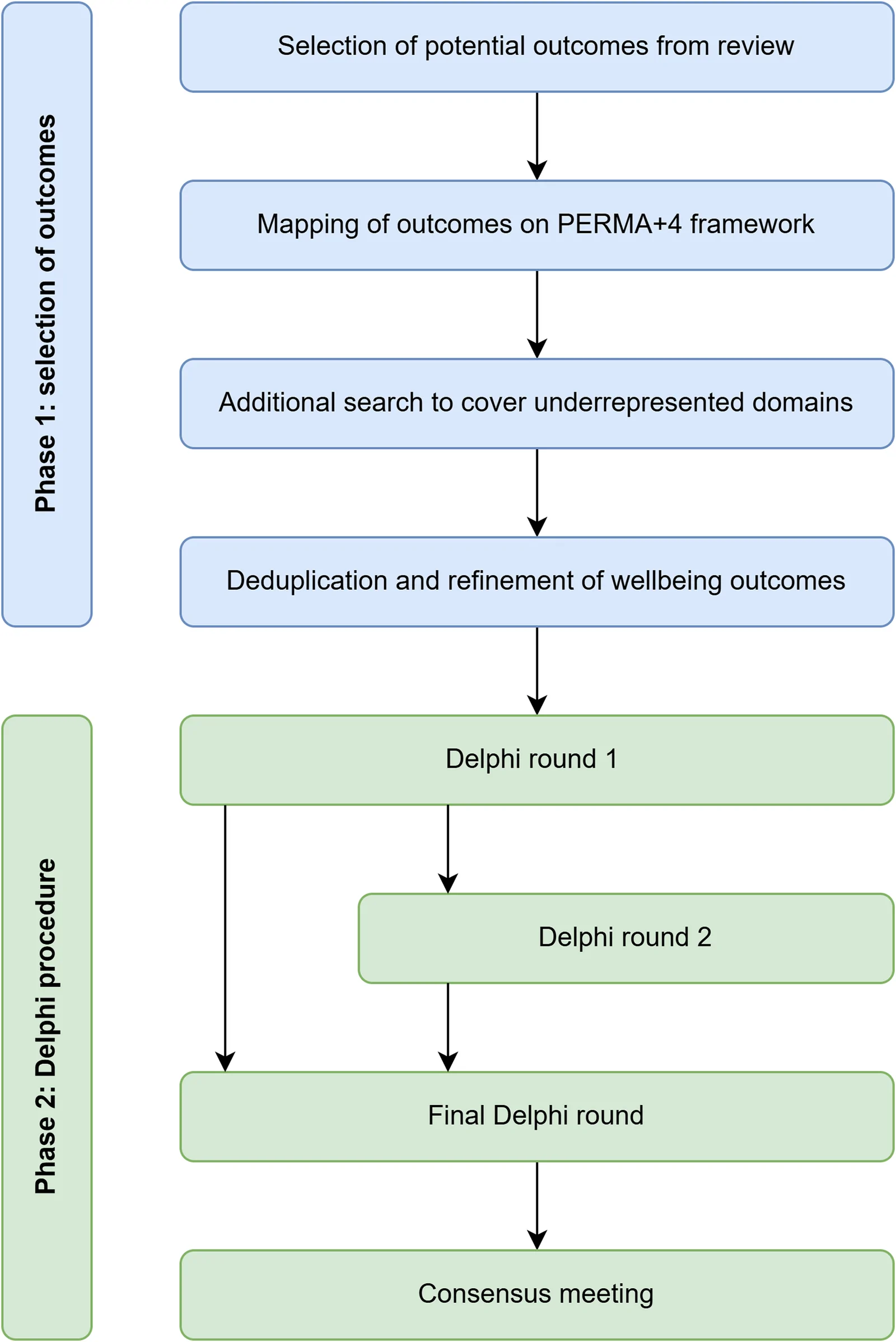
The flow of this study for the development of a core outcome set for wellbeing of the healthcare professionals. Candidate constructs were organised within the PERMA+4 framework (Positive emotions, Engagement, Relationships, Meaning, Accomplishment, plus Physical health, Mindset, Work environment, and Economic security).

Following completion of the survey rounds, the Steering Committee convened to review the results and formally confirm the final COS. A detailed description of the Delphi methodology and analytical approach is provided in the Supplementary material. The overall study flow is presented in Fig. 1.

### Data analysis

Analyses were primarily descriptive. For Delphi Rounds 1 and 2, results were summarised per construct as the proportion of participants rating 7–9 and 1–3, together with the median and interquartile range (IQR), in accordance with the predefined consensus criteria. “I don’t know” responses were treated as missing and excluded from agreement calculations, while percentages were calculated using the total number of panellists as the denominator. For Round 3, the percentage of participants ranking each construct in their top 10 was calculated, and constructs were ranked accordingly. The primary analysis excluded participants whose primary professional role was ’researcher’, to ensure that final prioritisation reflected the perspectives of practising healthcare professionals. Additional exploratory analyses were conducted to assess the robustness and contextual interpretation of the final ranking. These included weighted ranking analysis to account for unequal stakeholder group sizes, analyses by professional role and geo-economic region, domain-specific rankings within the PERMA+4 domains, and analyses of the contextual level at which constructs were considered most relevant, including the individual, team, and organisational levels. Full analytical details, including sensitivity analyses and weighted procedures, are provided in the Supplementary materials.

### Role of the funding source

The authors received no specific funding for this work.

## Results

### Protocol amendments

Three amendments to the registered protocol were made. First, consensus thresholds were refined after Round 1. The initially prespecified thresholds (≥70% of participants rating 7–9 and ≤15% rating 1–3) resulted in limited discrimination between constructs, as most items received uniformly high ratings. Prior to proceeding to Round 2, the inclusion criteria were therefore tightened to ≥85% rating 7–9 and <15% rating 1–3 to improve specificity and enhance prioritisation. A formal sensitivity analysis using the original thresholds was not performed, as this would not have informed subsequent rounds and would have substantially increased respondent burden by requiring re-rating of a large number of constructs.

Second, a third Delphi round was added. After Round 2, the number of constructs meeting inclusion criteria still exceeded the predefined maximum of ten outcomes for the final COS. A ranking round was therefore introduced to enable structured prioritisation and reduction to a feasible set. In this final round, added after discussion with the core research group, participants also indicated the contextual level (individual, team, organisational, system) at which constructs were considered most relevant, to support interpretability and implementation. Third, the criterion for adding newly suggested constructs was operationalised differently from the preregistered protocol. The protocol specified that constructs suggested by more than 1% of panellists would be considered for inclusion in the next round. Because the achieved panel size was smaller than anticipated, this threshold would have allowed constructs suggested by a single participant to be added. We, therefore, required newly suggested constructs to be mentioned by at least two panel members and to be conceptually distinct from constructs already included in the list. Further methodological details are provided in the Supplementary material (Appendix A).

### Development of an outcome list

**Fig. 2:**
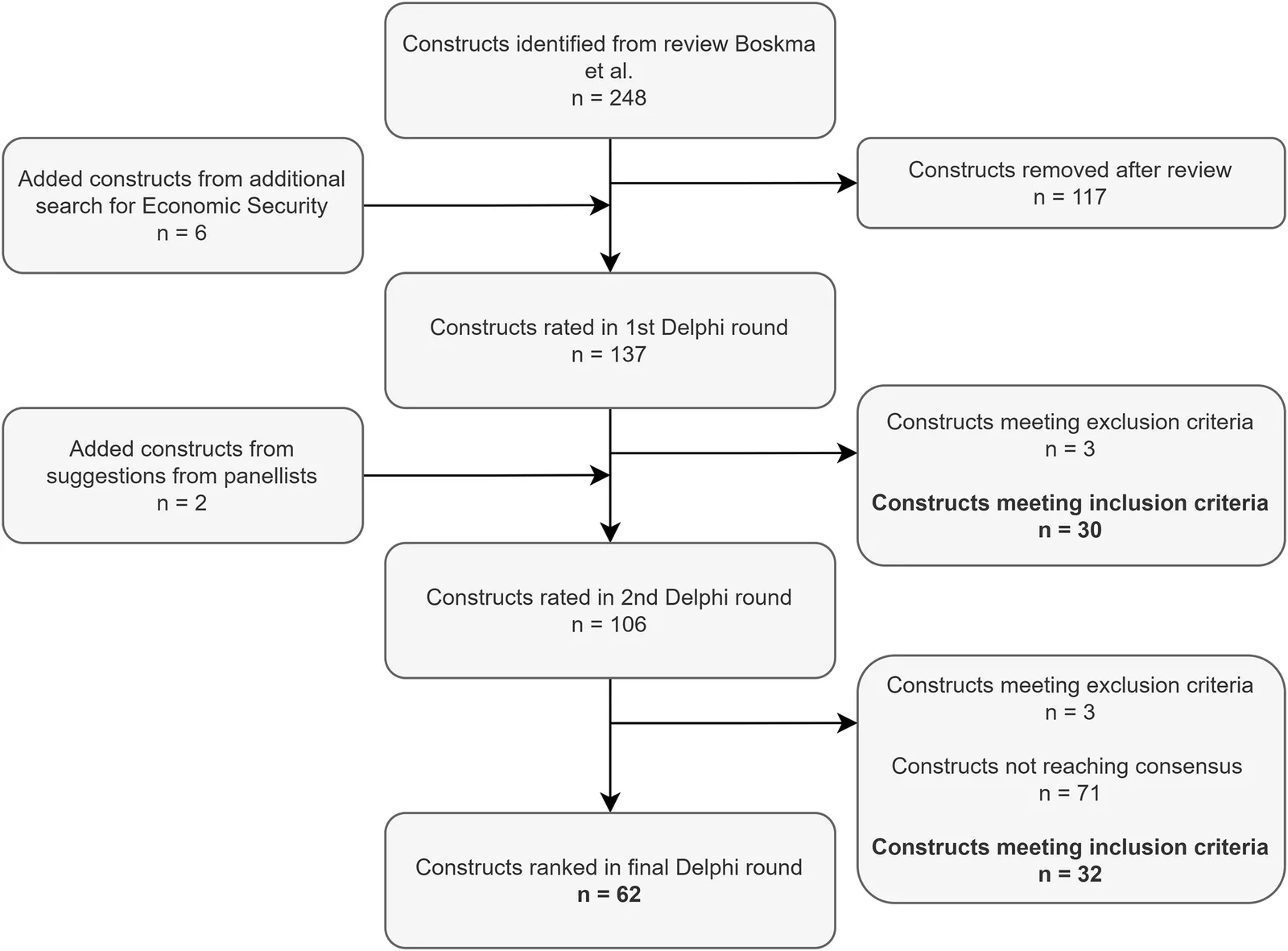
An overview of how constructs were selected throughout the whole Delphi process.

The scoping review by Boskma et al.^23^ identified 248 wellbeing constructs. The additional search addressing economic determinants yielded 55 records, of which 16 were assessed in full text. Six met inclusion criteria. This resulted in six additional economic constructs, generating a preliminary list of 254 constructs (Supplementary Table S3). Following screening, deduplication, and conceptual refinement, 137 unique constructs were retained for Delphi evaluation (Supplementary Table S4). These were distributed across the PERMA+4 domains as follows: Positive emotions (28), Engagement (12), Relationships (13), Meaning (6), Accomplishment (12), Physical health (13), Mindset (28), Environment (22), and Economic security (4). An overview of construct selection and refinement process is shown in Fig. 2.

### Participants

Across the three Delphi rounds, 57 (100% of invited panellists), 50 (87.7%) and 46 (80.7%) panel members participated, respectively. Participant characteristics remained stable across rounds (Table 1). Panellists participated from 20 countries across six continents. Detailed cross-tabulations of professional roles by geo-economic region are provided in Supplementary Table S5.

**Table 1:** Characteristics of panellists.

	Round 1	Round 2	Round 3
Participants (n)	57	50	46
Age (median age in years (IQR))	36 (32–47)	36 (32–47)	36 (32–48)
Gender			
Male (n (%))	30 (52.6)	27 (54.0)	26 (56.5)
Female (n (%))	26 (45.6)	22 (44.0)	19 (41.3)
Don’t want to disclose (n (%))	1 (1.8)	1 (2.0)	1 (2.2)
Professional background			
Physician (n (%))	19 (33.3)	15 (30.0)	14 (30.4)
Nurse (n (%))	14 (24.6)	14 (28.0)	13 (28.3)
Allied health professional (n (%))	6 (10.5)	6 (12.0)	6 (13.1)
Researcher (n (%))	14 (24.6)	12 (24.0)	10 (21.7)
Other (n (%))	4 (7.0)	3 (6.0)	3 (6.5)
Geo-economic region^a^			
High income (n (%))	31 (54.4)	27 (54.0)	24 (52.2)
Middle income (n (%))	12 (21.0)	10 (20.0)	9 (19.6)
Low-income (n (%))	14 (24.6)	13 (26.0)	13 (28.2)

IQR: interquartile range.

a Geo-economic region was defined according to World Bank income classifications.

### Delphi procedure

#### Round 1

Round 1 was conducted between July 27 and Sept 1, 2025. In round 1, panellists were presented the start set of 137 constructs. Thirty items met the inclusion criteria and three were excluded, 104 progressed to round 2. Based on panel member suggestions, two new constructs were added for the next round: Family wellbeing (mentioned by five panel members) and Professional equality (mentioned by two panel members). Detailed per item results and handling of suggestions are provided in Supplementary Tables S6 and S7.

#### Round 2

In round 2, conducted between Sept 25 and Oct 31, 2025, 106 constructs were presented for re-evaluation. 32 additional constructs met inclusion criteria, resulting in 62 constructs entering round 3. Detailed per item results and handling are provided in Supplementary Table S8.

#### Round 3 and development of core outcome set

**Fig. 3:**
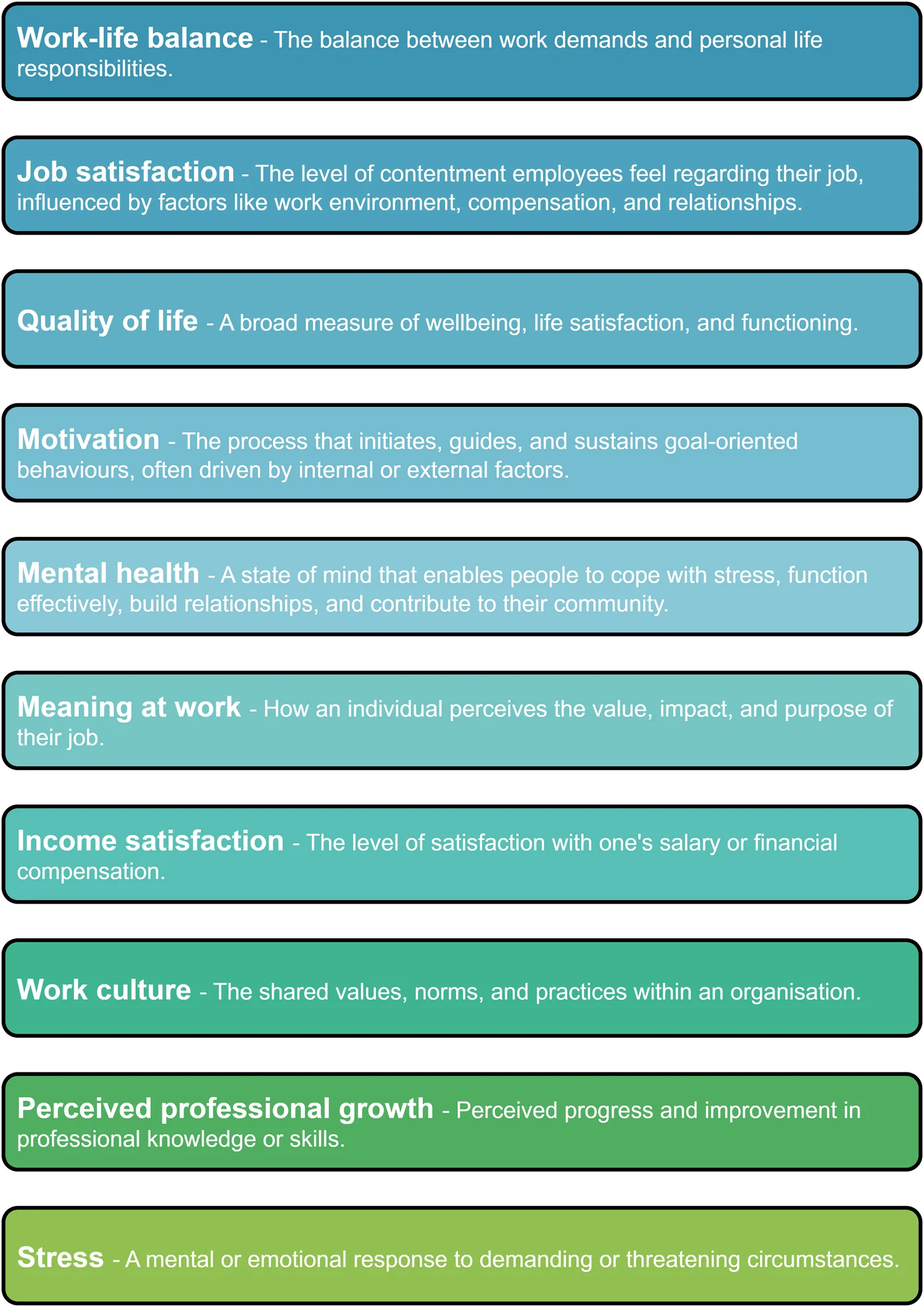
The final core outcome set.

**Fig. 4:**
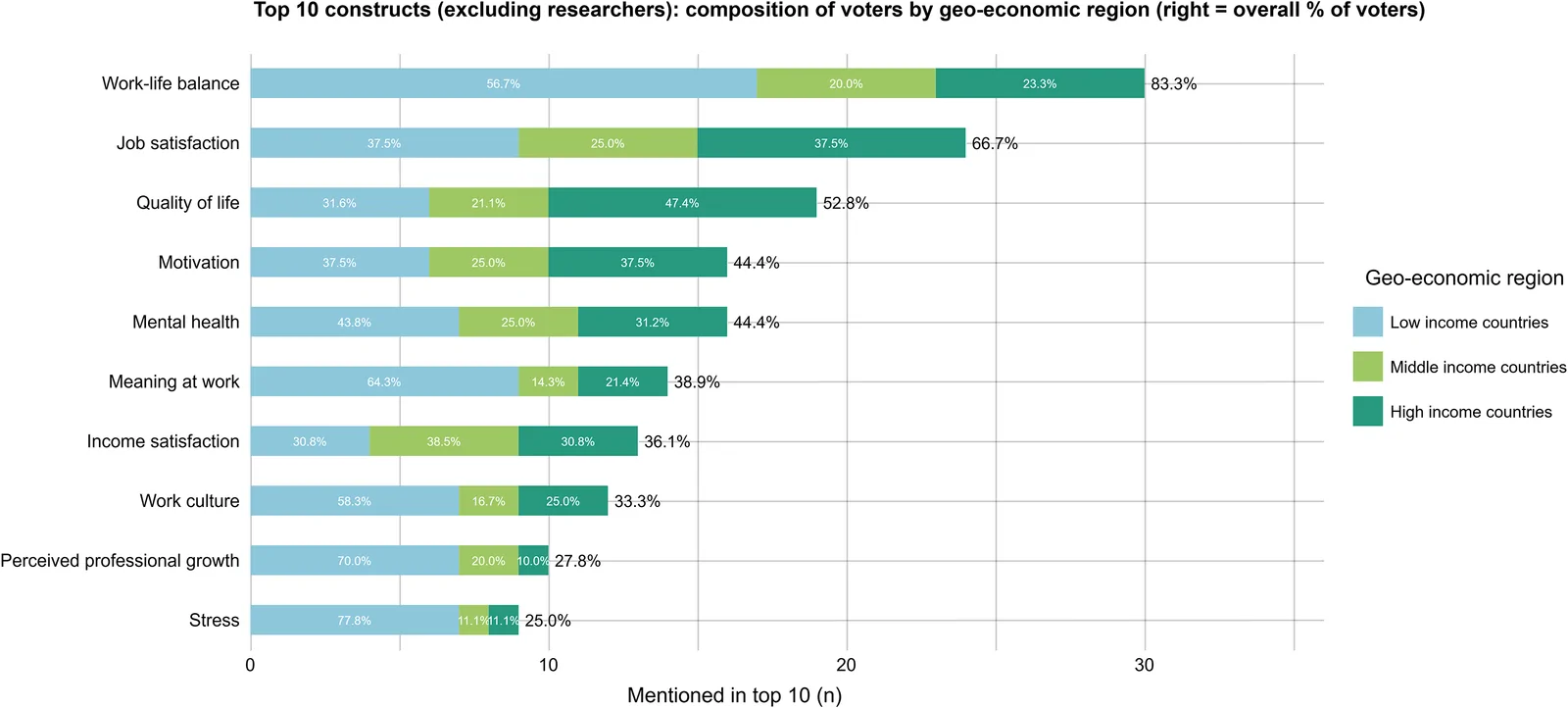
Composition of voters by geo-economic region for the core outcome set.

Round 3 was conducted between Nov 20, 2025, and Jan 7, 2026. Ranking of the 62 included constructs resulted in a final top-10 core outcome set (Fig. 3). The Steering Committee reviewed and formally endorsed the panel-derived ranking; no changes were made to the order of constructs or to the composition of the final COS. Work–life balance, job satisfaction, and quality of life were most frequently prioritised. The ranking of all 62 constructs is provided in Supplementary Table S9 (all panellists) and S10 (clinical panellists only). Fig. 4 presents an overview of the ten highest-ranked constructs with the distribution of voters across geo-economic regions for each selected construct. Each outcome was accompanied by a short working definition during the consensus process to ensure consistent interpretation (Supplementary Table S23). The top 10 constructs remained stable after a weighted ranking, correcting for stakeholder group size, only exchanging stress by satisfaction with career identity from this top 10 (Supplementary Tables S11 and S14). Results of this sensitivity analysis and analysis by professional role and geo-economic context of the constructs and core outcome set are presented in the Supplement (Appendix B: supplemental results).

Domain-specific analyses identified that constructs for job satisfaction, work–life balance, belongingness, mental health, quality of life, income satisfaction, and perceived organisational support were consistently prioritised (Supplementary Tables S18 and S19). Prioritisation differed by level of influence. At the individual level, job satisfaction and work–life balance received the highest weighted scores. Team-level rankings emphasised motivation, job satisfaction, and perceived support, whereas organisational-level priorities centred on motivation, burnout, and leadership-related constructs (Supplementary Fig. S14).

## Discussion

In this international modified Delphi study, we developed a ten-item core outcome set for healthcare professional wellbeing following three Delphi rounds. From 62 constructs evaluated in the final round, work–life balance, job satisfaction, and quality of life were most highly prioritised. The top ten remained stable after individual weighting of scores. After weighting for stakeholder group size, only one substitution was observed (Stress replaced by Career identity satisfaction). This shift suggests that professional identity may represent a particularly salient dimension of wellbeing across stakeholder groups, becoming more prominent when differences in group size are taken into account. Sensitivity and subgroup analyses yielded comparable results.

This COS provides a structured response to persistent outcome heterogeneity in HCP wellbeing research.^28^ Interventions are currently evaluated using highly diverse and non-overlapping endpoints, which limits comparability and weakens cumulative evidence synthesis.^14^ By defining a set of ten agreed outcomes, this study offers a shared measurement foundation that can improve interpretability of evaluations and support cross-context learning.

When mapped back to the PERMA+4 framework,^16^ the ten constructs in the COS span eight of the nine domains, leaving only Relationships unrepresented. From a theoretical perspective, this broad domain coverage reinforces the conceptual completeness of the COS. By encompassing nearly the full theoretical spectrum of PERMA+4, the COS reflects a conceptually comprehensive and multidimensional representation of HCP wellbeing. The absence of the Relationships domain is notable, as collegial support and positive team relations are often considered central to work pleasure.^29^ A possible explanation is that panellists perceived relational aspects less as outcomes in themselves and more as determinants or contextual conditions that shape wellbeing. When looking at the COS from another, more commonly used theoretical perspective, namely the Job Demands and Resources model,^30^ five of the six domains are represented, further underscoring that the found COS captures nearly the full spectrum of wellbeing. This Job Demands and Resources model might also help explain that constructs on Relationships function as a job resource, and in that way buffer the demands of the job, and are thus more a mediating pathway, rather than an outcome.

The content of our COS aligns with earlier, more narrowly scoped initiatives. A UK Delphi study among doctors in the NHS similarly identified job satisfaction and work–life balance as core outcomes.^17^ Convergence on these constructs across a single-system, profession-specific study and our international, multi-professional panel suggests that they represent stable anchor indicators of HCP wellbeing. At the same time, the broader professional and geo-economic representation in our study enhances generalisability and strengthens the applicability of the COS for international research and multinational implementation efforts. Our international and interprofessional panel inevitably brought together diverse healthcare realities. The resulting COS should therefore be understood as context-transcending in content, but context-sensitive in interpretation. While the selected outcomes represent shared priorities across settings, their meaning, determinants, and practical implications will vary depending on local structures, resources, and cultural norms.

Some constructs emphasised in workforce theory, such as autonomy or job control, were not prioritised into the final COS.^31^^,^^32^ This should not be interpreted as irrelevance. Autonomy may be embedded within higher-ranked outcomes (e.g., job satisfaction and work–life balance) and within contextual outcomes related to leadership and organisational support, where it becomes visible through downstream effects. Alternatively, panellists may conceptualise autonomy primarily as a determinant rather than an endpoint: a design feature to improve, rather than a final indicator of wellbeing. This interpretation is consistent with the Self-Determination Theory, in which autonomy is a basic psychological need that drives motivation and satisfaction, rather than a terminal outcome.^32^ In that sense, the present COS may be understood as a set of core endpoints, while determinant measures remain important for needs assessment, intervention selection, and explaining why outcomes improve—or do not improve—over time. A further consideration is that HCP wellbeing is also influenced by factors outside the workplace, such as family demands. These were not explicitly incorporated into the present COS, which may therefore be more reflective of work-related wellbeing than of the full broader life context in which wellbeing is experienced.

This study has several strengths. First, this study combined an evidence-informed and conceptually structured longlist with international, multi-professional stakeholder consensus. Second, constructs were clearly defined to support consistent interpretation, and retention across Delphi rounds was high, enhancing the stability of findings. Third, overall retention of participants was high (80.7%), supporting the stability of the consensus process. However, reasons for drop-out of participants were not collected, so we cannot determine whether attrition was related to survey burden, time constraints, loss of interest, or other factors. Finally, when importance ratings showed limited discrimination, a structured ranking round was introduced to ensure development of a feasible and implementable COS based on relative prioritisation.

Limitations should be acknowledged. First, the longlist was derived from a scoping review rather than a systematic review. This approach was considered appropriate because the aim was to map the breadth of constructs used to measure HCP wellbeing, rather than to evaluate intervention effects or associations. Nevertheless, relevant constructs may have been missed, particularly those reported in lower-visibility literature. We mitigated this by mapping constructs to the PERMA+4 framework, conducting an additional targeted search for the underrepresented Economic Security domain, and allowing participants to suggest additional constructs in Round 1. Second, recruitment proved more challenging than anticipated. This resulted in relatively low representation of allied health professionals and a predominance of participants from high-income regions, which may limit the global and fully interprofessional generalisability of the COS. However, because this was a Delphi consensus study, the panel was intended to capture relevant stakeholder perspectives rather than to be statistically representative of the global HCP workforce. Furthermore, the panel still included participants from middle- and low-income settings and a substantial representation of nurses, supporting the relevance of the COS across a range of healthcare contexts and professional perspectives. We initially aimed to include 90–120 participants to enable exploration of stakeholder- or region-specific COSs. This was not feasible within a reasonable timeframe, which limited the possibility to perform robust subgroup analyses. We therefore proceeded with a smaller but engaged panel (57 in round 1, with retention of 80.7%), resulting in a single overarching COS. The heterogeneity of the panel may have introduced differences in how constructs were interpreted and prioritised across professional roles, healthcare systems, resource settings, and cultural contexts. As a result, some subgroup-specific priorities may have been obscured in the overall ranking. However, exploring subgroup analyses nevertheless showed substantial overlap in prioritised constructs across professional subgroups and geo-economic regions, suggesting that the final COS captured a broadly shared set of priorities despite unequal subgroup sizes. These findings provide some reassurance, but should be interpreted cautiously because subgroup numbers were small. Third, the panel was relatively young, plausibly reflecting recruitment through digital professional networks. Age is an imperfect proxy for professional experience, but a younger panel may have placed greater weight on certain outcomes, as previous research among nurses has described generational differences in work values, including greater emphasis on work–life balance among younger generations.^33^ Although prioritisation was broadly consistent across subgroups, we cannot exclude an influence of the panel’s age profile on the relative ranking of constructs. Finally, the high ratings across many constructs limited differentiation based on absolute thresholds alone. This likely reflects alignment between the longlist and broadly valued dimensions of HCP wellbeing. The addition of a ranking round allowed meaningful prioritisation without compromising consensus.

For research and practice, this COS should be used as a core outcome framework in trials, implementation studies, and routine monitoring targeting HCP wellbeing. The core outcomes together define the central construct to be evaluated, but the COS does not prescribe a single universal primary endpoint or require the outcomes to be combined into a composite score. Individual studies should predefine how the COS will be operationalised, depending on the intervention’s hypothesised mechanism, the wellbeing problem being targeted, baseline relevance in the study population, stakeholder priorities, feasibility, and the measurement properties of available instruments. This approach preserves comparability without erasing context. Wellbeing is a broad and multidimensional construct, as reflected in the 254 candidate constructs identified in the prior scoping review. Some constructs represent broad outcome domains, whereas others are more context-specific or function primarily as determinants of those outcomes. The present COS should therefore not be seen as an exhaustive representation of HCP wellbeing, but as a set of broadly relevant core outcomes. Depending on the purpose and setting of a study, these outcomes may need to be complemented by local modules or determinant measures to capture context-specific variation and inform intervention design.

For settings with existing longitudinal wellbeing measurement systems, adoption of the COS should not require abrupt discontinuation of established indicators. Instead, current measures can be mapped onto the COS to identify which core outcomes are already captured and where gaps remain. Missing COS outcomes can then be added alongside existing measures, allowing continuity of time-series data while gradually improving comparability with future studies. Where parallel measurement is feasible, it may also support calibration between historical indicators and COS-based measurement.

The next methodological step is development of a core outcome measurement set (COMS) that recommends instruments for each outcome based on reliability, validity, responsiveness, feasibility, cross-cultural measurement properties, and administrative burden. Implementation also requires clarity about who measures what, when, and at what level. Our level-specific findings support a layered approach. Individual-level outcomes can inform supportive services, team-level outcomes can guide local improvement cycles, and organisational-level outcomes can support governance and leadership interventions. To avoid shifting responsibility to individuals, organisations should explicitly own the measurement process, ensure feedback loops, and minimise respondent burden through proportionate measurement frequency and integration with routine data where appropriate.

In conclusion, this COS provides an internationally derived, implementable set of outcomes for HCP wellbeing. Although effective interventions exist, the field has lacked the shared measurement foundation to establish what works, for whom, and under what conditions. By providing an internationally derived, implementable set of outcomes, it creates the basic condition for the field to move forward: a common language that makes studies comparable, evidence accumulable, and improvement measurable. This study establishes consensus on which outcomes should be measured. It does not itself evaluate implementation or demonstrate effects on workforce outcomes. Realising the potential benefits will depend on adoption of the COS, selection of validated instruments (a forthcoming core outcome measurement set), and concurrent measurement of key determinants. With these steps, a shared outcome foundation can support efforts to sustain a workforce that health systems can ill afford to lose.

## Contributors

T. Bazuin: Conceptualisation, Data curation, Formal analysis, Investigation, Methodology, Project administration, Validation, Visualisation, Writing–original draft. S.F.C. Mugge: Conceptualisation, Data curation, Formal analysis, Investigation, Methodology, Project administration, Validation, Visualisation, Writing–original draft. A.C.P. Boskma: Conceptualisation, Data curation, Methodology, Validation, Writing–review and editing. J. Lopez-Alcalde: Conceptualisation; Methodology, Writing–review and editing. D.A. Dongelmans: Conceptualisation, Methodology, Supervision, Writing–review and editing. L. Hooft: Conceptualisation, Methodology, Supervision, Writing–review and editing. M.J. van der Laan: Conceptualisation, Methodology, Supervision, Writing–review and editing. All authors had full access to the study data, read and approved the final version of the manuscript, and accept responsibility for the decision to submit for publication. S.F.C. Mugge and T. Bazuin have accessed and verified the data. The CROWN Steering Committee reviewed the study design, monitored the Delphi process, and reviewed and endorsed the final core outcome set. The CROWN Panellist Group participated in the Delphi surveys and construct prioritisation.

## Data sharing statement

Data collected in this study can be made available upon reasonable request, directed to the corresponding author. A signed data access agreement will be required. The study protocol is available on the Open Science Framework (https://osf.io/9c5pr).

## Declaration of interests

No conflicts of interests were reported by the authors.
