# Supplementary material for: A core outcome set for the wellbeing of healthcare professionals: an international Delphi consensus study
Source: eClinicalMedicine. 2026 Aug 17;99:104145. doi: 10.1016/j.eclinm.2026.104145 (PMC13616565; doi:10.1016/j.eclinm.2026.104145)
Supplement: Supplementary file 1 [file mmc1.docx]

A Core Outcome Set for the Wellbeing of Healthcare Professionals: An International Delphi Consensus Study – supplementary material

This supplement provides additional methodological detail, detailed Delphi results, and reporting checklists.

# Contents

[Supplemental Tables	4](#_Toc235783562)

[Supplemental Table S1. COS-STAD recommendations	4](#_Toc235783563)

[Supplemental Table S2. Search string additional search economic constructs	5](#_Toc235783564)

[Supplemental Table S3. Raw outcomes from literature search (including additional search on economic constructs (n= 254))	6](#_Toc235783565)

[Supplemental Table S4. Initial start-set (n=137), including PERMA+4 mapping	14](#_Toc235783566)

[Supplemental Table S5. Composition of panel members across professional roles and geo-economic regions in round 3	19](#_Toc235783567)

[Supplemental Table S6. Results per item from Delphi round 1	20](#_Toc235783568)

[Supplemental Table S7. Suggestions from round 1 and actions on it	24](#_Toc235783569)

[Supplemental Table S8. Results per item from Delphi round 2	26](#_Toc235783570)

[Supplemental Table S9. Results from Delphi round 3, all panel members	29](#_Toc235783571)

[Supplemental Table S10. Results from Delphi round 3, clinical panel members	31](#_Toc235783572)

[Supplemental Table S11. Weighted ranking of constructs, all panel members	33](#_Toc235783573)

[Supplemental Table S12. Weighted ranking of constructs, clinical panel members	35](#_Toc235783574)

[Supplemental Table S13. Weighted ranking of constructs according to REPS, all panel members	37](#_Toc235783575)

[Supplemental Table S14. Weighted ranking of constructs according to REPS, clinical panel members	39](#_Toc235783576)

[Supplemental Table S15. Top 10 constructs per geo-economic stakeholder group (all panel members)	41](#_Toc235783577)

[Supplemental Table S16. Top 10 constructs per geo-economic stakeholder group (clinical panel members)	42](#_Toc235783578)

[Supplemental Table S17. Top 10 constructs within professional role	43](#_Toc235783579)

[Supplemental Table S18. Top 3 ranked constructs within PERMA+4 domains (including all panel members)	44](#_Toc235783580)

[Supplemental Table S19. Top 3 ranked constructs within PERMA+4 domains (excluding researchers)	45](#_Toc235783581)

[Supplemental Table S20. Top 5 ranked constructs within individual, team or organizational level (all panel members)	46](#_Toc235783582)

[Supplemental Table S21. Top 5 ranked constructs within individual, team or organizational level (clinical panel members)	47](#_Toc235783583)

[Supplemental Table S22. COS-STAR Statement	48](#_Toc235783584)

[Supplemental Table S23. Final COS items and definitions	50](#_Toc235783585)

[Supplemental Figures	51](#_Toc235783586)

[Supplemental Figure S1. Consensus process for developing the primary COS	51](#_Toc235783587)

[Supplemental Figure S2. Overview of all responses Delphi Round 1	52](#_Toc235783588)

[Supplemental Figure S3. Responses per item from Delphi round 1	53](#_Toc235783589)

[Supplemental Figure S4. Overview of all responses Delphi Round 2	71](#_Toc235783590)

[Supplemental Figure S5. Responses per item from Delphi round 2	72](#_Toc235783591)

[Supplemental Figure S6. Top 10 constructs when including all panel members	86](#_Toc235783592)

[Supplemental Figure S7. Top 10 constructs among clinical panel members	87](#_Toc235783593)

[Supplemental Figure S8. Voters by geo-economic group on overall top 10	88](#_Toc235783594)

[Supplemental Figure S9. Voters by geo-economic region on the clinical top 10	89](#_Toc235783595)

[Supplemental Figure S10. Within geo-economic group prioritization of the overall top 10	90](#_Toc235783596)

[Supplemental Figure S11. Within geo-economic group prioritization of the clinical top 10	91](#_Toc235783597)

[Supplemental Figure S12. Within professional-role prioritization of the overall top 10	92](#_Toc235783598)

[Supplemental Figure S13. Within professional-role prioritization of the clinical top 10	93](#_Toc235783599)

[Supplemental Figure S14. Prioritization of constructs across levels of all panel members	94](#_Toc235783600)

[Supplemental Figure S15. Prioritization of constructs across levels excluding researchers	95](#_Toc235783601)

[Appendices	96](#_Toc235783602)

[Appendix A: Additional methodological details	96](#_Toc235783603)

[1.1 Governance and stakeholder involvement	96](#_Toc235783604)

[1.2 Developing the initial core outcome set	96](#_Toc235783605)

[1.3 Delphi survey design and consensus criteria	97](#_Toc235783606)

[1.4 Delphi rounds	98](#_Toc235783607)

[Appendix B: Supplemental results	100](#_Toc235783608)

[2.1 Participant flow and composition	100](#_Toc235783609)

[2.2 Delphi results by round	100](#_Toc235783610)

[2.3 Subgroup and secondary analysis	100](#_Toc235783611)

[Appendix C. List of Steering committee members	102](#_Toc235783612)

[Appendix D. List of Panel members that contributed to >1 Delphi round	103](#_Toc235783613)

[Appendix E. Decision of medical ethics committee	104](#_Toc235783614)

# Supplemental Tables

## Supplemental Table S1. COS-STAD recommendations

| **Domain** | **Number** | **Methodology** | **Application** |
| --- | --- | --- | --- |
| Scope specification | 1 | The research or practice setting(s) in which the COS is to be applied | Wellbeing in healthcare professionals |
|  | 2 | The health condition(s) covered by the COS | Wellbeing |
|  | 3 | The population(s) covered by the COS | Healthcare professionals |
|  | 4 | The intervention(s) covered by the COS | None. |
| Stakeholders involved | 5 | Those who will use the COS in research | Healthcare professionals, researchers, policy makers in healthcare |
|  | 6 | Healthcare professionals with experience of patients with the condition | Healthcare workers themselves |
|  | 7 | Patients with the condition or their representatives | NA |
| Consensus process | 8 | The initial list of outcomes considered both healthcare professionals’ and patients’ views. | NA |
|  | 9 | A scoring process and consensus definition were described a priori. | Present, described in methods. |
|  | 10 | Criteria for including/dropping/adding outcomes were described a priori. | Present, described in methods. |
|  | 11 | Care was taken to avoid ambiguity of language used in the list of outcomes. | Present, through use of definitions and carefully phrasing in collaboration with SC. |

## Supplemental Table S2. Search string additional search economic constructs

| **#** | **Searches** | **Results** |
| --- | --- | --- |
| 1 | ("Healthcare professionals" or Caregivers or "Healthcare providers" or Practitioners or Doctor or nurse? or physician? or resident? or "healthcare worker" or "health staff").ti. or exp *"physicians"/ or exp *"Medical staff"/ or *"Residents"/ or exp *"Nurses"/ or exp *"Nursing Staff"/ | 489520 |
| 2 | (poverty or income* or wages or ((housing or living or financial or economic) adj2 (status or standard*))).ti,ab,kf. | 216659 |
| 3 | income/ or "salaries and fringe benefits"/ | 52024 |
| 4 | Personnel Turnover/ or exp Job Satisfaction/ or (((job or work) adj3 (satisfaction or culture or environment)) or work-load or workload or (sick adj2 leave) or absenteeism or presenteeism or ((turnover or leave) adj3 intention) or ((Employee or Personnel) adj3 (Turnover* or Retention*))).ti,ab,kf. or exp Job Satisfaction/ or exp Organizational Culture/ or exp Workload/ or exp Absenteeism/ or exp Presenteeism/ or exp Burnout, Psychological/ or exp Personal Satisfaction/ or (((work or job) adj3 satisfaction) or workload or well-being or fulfilment or burnout or thriving).ti,ab,kf. or Work-Life Balance/ or exp Sick Leave/ | 275352 |
| 5 | review.pt. | 3037894 |
| 6 | 2 or 3 | 244183 |
| 7 | 1 and 6 and 4 and 5 | 102 |
| 8 | limit 7 to yr=''2011 -Current'' | 55 |

## **Supplemental Table S3.** Raw outcomes from literature search (including additional search on economic constructs (n= 254))

| Nr | Item | Decision | Explanation |
| --- | --- | --- | --- |
| 1 | Ability to work | Rewrite | Rewritten to "perceived ability to work" |
| 2 | Absenteeism and performance | Rewrite | Absenteisme is a relevant outcome, but performance is more of a predictor. Rewritten as "absenteeism" |
| 3 | Abuse | Exclude | Functions more as a personal stressor than an outcome |
| 4 | Abusive supervision | Exclude | Functions more as a personal stressor than an outcome |
| 5 | Activity | Exclude | Does not represent a meaningful or measurable outcome. |
| 6 | Addiction to work | Exclude | Excluded as it is better classified as a predictor, not an outcome. |
| 7 | Adequate allowance payments | Exclude | Overlaps with reward |
| 8 | Aggressiveness | Include | As part of a job demand |
| 9 | Alarm fatigue | Exclude | Excluded as it is context-specific and considered an effect modifier. |
| 10 | Alexithymia | Include | Personal and context relevant stressor |
| 11 | Altruism | Include |  |
| 12 | Anxiety and self-confidence | Rewrite | To anxiety and "self-confidence in professional role" |
| 13 | Assertiveness | Include |  |
| 14 | Attachment type | Exclude |  |
| 15 | Attitude to risks | Exclude |  |
| 16 | Attitude towards death | Exclude | It reflects an attitude, not an outcome. |
| 17 | Autonomy | Include |  |
| 18 | Avoidance behaviour, intrusive memories | Rewrite | Rewritten to two separate constructs. Intrusive memories are valid outcomes related to psychological burden or PTSD. Avoidance behaviour, however, is better viewed as a coping style and not a primary outcome. |
| 19 | Balance between income and workload | Rewrite | Relevant if reframed as 'fairness of compensation relative to workload'. |
| 20 | Behaviour and mood | Exclude | Excluded due to vagueness and overlap; better categorized as predictors. |
| 21 | Beliefs about mastery and performance mindsets | Exclude | Excluded because it is better understood as an effect modifier. |
| 22 | Belongingness | Include |  |
| 23 | Boundary spanning | Exclude | Due to its modifying role in interprofessional contexts. |
| 24 | Breastfeeding support | Exclude | Considered a provision or service, not an outcome. |
| 25 | Building satisfaction | Include |  |
| 26 | Bullying | Include |  |
| 27 | Bureaucratic demands | Rewrite | Important but must be framed as a burden. Rewritten as 'bureaucracy-related stress'. |
| 28 | Burnout | Include |  |
| 29 | Calling | Include |  |
| 30 | Career identity | Rewrite | Too vague in original form. Rewritten as 'satisfaction with career identity'. |
| 31 | Caring behaviours | Exclude | Excluded as a contextual factor, not a measurable outcome. |
| 32 | Caring efficacy | Include |  |
| 33 | Characteristics of different generation of nurses | Exclude | Due to being too specific and not broadly applicable. |
| 34 | Climate | Include |  |
| 35 | Cognitive appraisals | Exclude | Doubtful. Unclear how this construct would be defined and measured. |
| 36 | Coherence | Exclude | As it is a determinant of wellbeing, not an outcome itself. |
| 37 | Collaboration | Rewrite | Relevant, but currently too vague. Rewritten as 'experienced collaboration with colleagues'. |
| 38 | Colleague support | Exclude | As it modifies experience rather than representing an outcome. |
| 39 | Collectivist orientation | Exclude | As a cultural or modifying influence, not an outcome. |
| 40 | Collegial nurse/doctor relationship | Exclude | Due to being a relational modifier, not an outcome. |
| 41 | Commitment | Rewrite | Too broad; rewritten and reframed as 'professional commitment'. |
| 42 | Communication skills | Exclude | Because it is a skill, not an outcome measure. |
| 43 | Compassion | Exclude | Important motivational factor, though too broad. Self-compassion already included. |
| 44 | Compassion fatigue | Include |  |
| 45 | Confidence in communication | Exclude | Considered an effect modifier rather than an outcome. |
| 46 | Conflict | Include |  |
| 47 | Conflict management | Exclude | As it refers to relational processes, not outcomes. |
| 48 | Coping | Include |  |
| 49 | Covid-19 obsession | Exclude | Due to being highly context-specific. |
| 50 | Cultural awareness | Exclude | As it functions more as a cultural modifier, not a direct outcome. |
| 51 | Culture | Rewrite | Rewrite to Work culture for clear distinguishment. |
| 52 | Cynicism | Include |  |
| 53 | Death distress | Rewrite | Relevant if reframed as 'death-related anxiety'. |
| 54 | Death obsession | Exclude | As it is not defined as a measurable outcome. |
| 55 | Decision fatigue | Rewrite | Too vague. Rewritten as 'cognitive overload at work' to improve specificity. |
| 56 | Decision latitude | Exclude | Being too vague and classified as an effect modifier. |
| 57 | Decisional involvement | Exclude | Due to its modifying nature and lack of outcome status. |
| 58 | Demands, control support | Exclude | Due to vagueness of both concepts. |
| 59 | Depression, anxiety, stress | Rewrite | This entry combines three distinct constructs. While each is a valid outcome, they should not be grouped together as a single measure. |
| 60 | Deprivation | Exclude | As it is better understood as an effect modifier. |
| 61 | Difficulties with end-of-life care | Exclude | Not representing a distinct or measurable outcome. |
| 62 | Disability/consequence of mental health issues | Rewrite | Appears relevant when reframed as 'perceived mental health-related impairment'. |
| 63 | Discrimination/harassment/abuse | Rewrite | Rewritten as 'distress from workplace harassment' to isolate the most impactful construct. |
| 64 | Dissatisfaction | Exclude | Because it is essentially the inverse of job satisfaction, which is already included. |
| 65 | Distractions | Exclude | Because it is a situational factor, not a measurable outcome. |
| 66 | Doctor-patient relationship | Exclude | Because the relationship is not itself an outcome. |
| 67 | Documentation burden | Include |  |
| 68 | Eating disorders | Exclude | Being too context-specific. |
| 69 | Effort reward | Rewrite | Relevant. Rewritten as 'perceived balance between effort and reward'. |
| 70 | Emotion regulation | Rewrite | Relevant if framed as difficulty managing emotions. Rewritten as 'difficulty in emotion regulation'. |
| 71 | Emotional exhaustion | Include |  |
| 72 | Emotional exhaustion and depersonalization | Exclude | Emotional exhaustion is already included; depersonalization excluded. |
| 73 | Emotional Intelligence | Include |  |
| 74 | Emotional labour | Exclude | It is considered an effect modifier rather than a direct outcome. |
| 75 | Emotions towards patients | Exclude | It is a modifying emotional attitude, not an outcome. |
| 76 | Empathy | Exclude | Because empathy is a skill, not a measurable outcome. |
| 77 | Empowerment | Exclude | Being overly vague and overarching in scope. |
| 78 | Engagement | Rewrite | Rewritten due to vague context; reframed as 'work engagement'. |
| 79 | Enthusiasm | Include |  |
| 80 | Expended energy | Exclude | For being vague and poorly defined. |
| 81 | Experiences disruptive behaviours | Rewrite | Relevant, rewritten as 'distress related to aggressive or disruptive behaviours'. |
| 82 | Exposed to patient death | Exclude | Already covered under 'death distress'. |
| 83 | Extra-role behaviour | Exclude | Because it describes behavior, not an outcome. |
| 84 | Fatigue | Include |  |
| 85 | Fear | Include |  |
| 86 | Fear of compassion | Exclude | Excluded and merged conceptually with 'Fear' due to overlapping content. |
| 87 | Fear of COVID-19 | Exclude | Excluded and conceptually merged with 'Fear'; too specific and overlapping. |
| 88 | Feeling as a victim | Exclude | Due to lack of conceptual clarity and outcome definition. |
| 89 | Feeling of control | Include |  |
| 90 | Feeling prepared | Include |  |
| 91 | Flourishing | Include |  |
| 92 | Flow | Include |  |
| 93 | Forgetting intentions | Exclude | Being conceptually vague and unclear. |
| 94 | Functional status | Include |  |
| 95 | General health | Rewrite | Too general. Rewritten as 'self-rated health'. |
| 96 | Goal orientation | Include |  |
| 97 | Gratitude | Rewrite | Potentially protective factor. Rewritten as 'experienced gratitude'. |
| 98 | Grief | Include |  |
| 99 | Grit | Include |  |
| 100 | Happiness | Include |  |
| 101 | Hcp-patient relationship | Include |  |
| 102 | Healthy lifestyle behaviours | Exclude | As these behaviors are predictors or risk factors, not outcomes. |
| 103 | High performance work systems (HPWS) | Exclude | As the concept is vague and not outcome-oriented. |
| 104 | Hope | Include |  |
| 105 | Housing | Exclude | As housing is not considered a relevant outcome. |
| 106 | Humanism & Professionalism | Exclude | Neither concept represents a measurable outcome. |
| 107 | Imposter syndrome | Rewrite | Too vague. Rewritten as 'perceived professional inadequacy'. |
| 108 | Incivility | Exclude | As it is considered an effect modifier, not an outcome. |
| 109 | Income satisfaction | Include |  |
| 110 | Innovativeness | Exclude | Because the degree of innovativeness is not an outcome. |
| 111 | Insurance | Exclude | Not a measurable wellbeing outcome. |
| 112 | Intention to leave | Include |  |
| 113 | Interpersonal behaviour | Include |  |
| 114 | Intimidation | Include |  |
| 115 | Intuition | Exclude | Represents an internal feeling, not a measurable outcome. |
| 116 | Job characteristics | Exclude | As it is not defined as a measurable outcome. |
| 117 | Job control | Include |  |
| 118 | Job crafting | Include |  |
| 119 | Job demands | Exclude | Excluded due to lack of clarity and specificity. |
| 120 | Job fit | Include |  |
| 121 | Job insecurity | Include |  |
| 122 | Job performance | Exclude | Already covered under ‘satisfaction with performance’ |
| 123 | Job preferences | Exclude | Because it reflects preference, not a measurable outcome. |
| 124 | Job resources | Exclude | Due to vague formulation and lack of outcome focus. |
| 125 | Job resources and job demands | Exclude | As both terms are too vague to classify as outcomes. |
| 126 | Job rotation | Include |  |
| 127 | Job satisfaction | Include |  |
| 128 | Job satisfaction & psychosocial work environment | Exclude | Due to overlap and the vague definition of 'psychosocial work environment'. |
| 129 | Job satisfaction/wellbeing | Exclude | Due to redundancy and lack of specificity in combining two broad concepts. |
| 130 | Knowledge and practices of COVID-19 | Exclude | As it is considered an effect modifier related to context-specific knowledge. |
| 131 | Leadership | Exclude | As it functions as an effect modifier, not an outcome. |
| 13 | Leadership needs | Exclude | Because it is an effect modifier rather than a standalone outcome. |
| 133 | Lifelong learning | Include |  |
| 134 | Listening | Exclude | It is a skill, not a measurable outcome. |
| 135 | Loneliness | Include |  |
| 136 | Management and leadership | Rewrite | Combines two elements. Rewritten as 'perceived quality of leadership'. |
| 137 | Meaning at work | Include |  |
| 138 | Measure beliefs and actions in health advocacy in nursing | Exclude | Reflects beliefs, not a component of wellbeing. |
| 139 | Mental health | Include |  |
| 140 | Mentorship | Include |  |
| 141 | Mindfulness | Include |  |
| 142 | Mood | Include |  |
| 143 | Moral courage | Exclude | Functions more as a precursor than an outcome. |
| 144 | Moral disengagement | Exclude | Classified as a cognitive mechanism, not outcome. |
| 145 | Moral distress | Include |  |
| 146 | Moral elevation | Exclude | Due to limited measurability and overlap with modifiers. |
| 147 | Moral injury | Exclude | Could overlap with 'moral resilience', which is already included. |
| 148 | Moral resilience | Include |  |
| 149 | Moral sensitivity | Exclude | Considered an effect modifier rather than outcome. |
| 150 | Motivation | Include |  |
| 151 | Nature of the exchange relationship with the organization | Exclude | Due to vagueness and its role as a contextual factor. |
| 152 | Need for recovery | Include |  |
| 153 | Neglect of work | Include |  |
| 154 | Nurse experience | Rewrite | Relevant if reframed in a broader context. Rewritten as 'professional experience'. |
| 155 | Nursing governance | Include |  |
| 156 | Nursing image | Exclude | Excluded as it is a symbolic or contextual construct, not an outcome. |
| 157 | Occupational disidentification | Include |  |
| 158 | Optimism | Include |  |
| 159 | Organisational support | Rewrite | Rewritten as 'perceived organizational support'. |
| 160 | Organizational communication satisfaction | Rewrite | Rewritten as 'satisfaction with organizational communication style'. |
| 161 | Organizational constraints | Include |  |
| 162 | Organizational justice | Rewrite | Rewritten as 'perceived fairness in workplace'. |
| 163 | Organizational trust | Exclude | Due to vagueness and conceptual ambiguity. |
| 164 | Pain | Include |  |
| 165 | Participation in decision making | Include |  |
| 166 | Patient-to-nurse ratio | Rewrite | rewritten to patient-to-HCP ratio |
| 167 | Perceptions of organizational politics | Include |  |
| 168 | Personal development | Exclude | ; already included elsewhere. |
| 169 | Personality traits | Include |  |
| 170 | Posttraumatic growth | Exclude | Excluded as it is too vague and functions more as an effect modifier. |
| 171 | Presenteeism | Include |  |
| 172 | Procedural justice | Include |  |
| 173 | Professional benefit | Exclude | Due to lack of clarity and outcome relevance. |
| 174 | Professional development | Rewrite | Rewritten as 'perceived professional growth' to clarify the construct as a subjective outcome. |
| 175 | Professional fulfilment | Include |  |
| 176 | Professional identity | Include |  |
| 177 | Professional skill at using better knowledge for decision making | Exclude | As it is a skill or predictor, not an outcome. |
| 178 | professional state | Exclude | Due to unclear definition and lack of outcome specificity. |
| 179 | Professional values | Include |  |
| 180 | Psychological attachment | Exclude | It modifies psychological connection but is not an outcome. |
| 181 | Psychological capital | Exclude | Being too broad and not outcome-specific. |
| 182 | Psychological distress and somatic complaints | Rewrite | Both are important, but should be treated separately: "psychological distress" and "somatic complaints". |
| 183 | Psychological flexibility | Include |  |
| 184 | Psychological health status | Exclude | Lack of conceptual clarity. |
| 185 | Psychological impact | Exclude | Due to being conceptually vague and imprecise. |
| 186 | Psychological Need Satisfaction | Include |  |
| 187 | PTSD | Include |  |
| 188 | Quality of life | Include |  |
| 189 | Reactions to uncertainty | Exclude | Doubtful. Lacks clarity as a defined outcome construct. |
| 190 | Readjustment/reintegration | Exclude | No clearly defined or measurable outcomes. |
| 191 | Regret | Rewrite | Relevant if reframed as 'work-related regret'. |
| 192 | Relational dimension of work and its psychological effects in the nursing profession | Exclude | Due to complexity and vague formulation. |
| 193 | Relationships | Rewrite | Too vague. Rewritten as 'social connectedness at work' to emphasize work-related relationships. |
| 194 | Religious involvement | Exclude | Because it reflects a worldview, not a measurable outcome. |
| 195 | Religiousness/Spirituality | Exclude | Spirituality is already addressed as an included outcome. |
| 196 | Resilience | Include |  |
| 197 | Rewarding | Include |  |
| 198 | Role transition | Rewrite | Too vague. Rewritten as 'stress associated with role transitioning'. |
| 199 | Rumination | Include |  |
| 200 | Satisfaction with compensation model | Include |  |
| 201 | Satisfaction with performance | Include |  |
| 202 | Satisfaction with supervisor | Include |  |
| 203 | Self-care | Include |  |
| 204 | Self-compassion | Include |  |
| 205 | Self-concept | Exclude | Due to vagueness and its role as a predictor rather than an outcome. |
| 206 | Self-confidence | Rewrite | Rewritten as 'self-confidence in professional role'. |
| 207 | Self-efficacy | Include |  |
| 208 | Self-esteem | Include |  |
| 209 | Self-evaluation | Exclude | As it is overly broad and non-specific, already represented in self-efficacy |
| 210 | Sense of coherence | Include |  |
| 211 | Shame/guilt | Exclude | These are primarily predictors or effect modifiers, not standalone outcomes. |
| 212 | Shared vision / goals | Exclude | Because it functions as an effect modifier. |
| 213 | Shift work disorder | Exclude | Being too specific and difficult to generalize. |
| 214 | Sickness absence | Exclude | Absenteeism is already included elsewhere. |
| 215 | Skin lesions related to personal protective equipment | Exclude | Due to its highly specific and contextual nature. |
| 216 | Sleep | Include |  |
| 217 | Social capital | Exclude | due to its structural modifying nature, represented in other constructs |
| 218 | Social desirability | Exclude | Not a measurable outcome. |
| 219 | Social functioning | Include |  |
| 220 | Social responsibility of the hospital | Exclude | It reflects an organizational trait rather than a wellbeing outcome. |
| 221 | Social support | Exclude | It modifies context and is not a distinct outcome. |
| 222 | Spirituality | Rewrite | Rewritten as 'spiritual wellbeing'. |
| 223 | Staff perceptions | Exclude | Due to vagueness and conceptual ambiguity. |
| 224 | Stamina | Rewrite | Relevant when framed as 'work-related energy'. |
| 225 | Stigma | Exclude | Functions more as a modifying factor than a measurable outcome. |
| 226 | Suicidal ideation | Exclude | Not considered a measurable outcome. |
| 227 | Supervisory support | Exclude | It is a contextual determinant, not an outcome. |
| 228 | Support | Rewrite | Too vague. Rewritten as 'perceived support from colleagues'. |
| 229 | Team interaction | Exclude | Due to being a team-level process variable, not an outcome. |
| 230 | Team vitality | Include |  |
| 231 | Teamwork | Exclude | Represents a process, not an outcome. |
| 232 | Tolerance for ambiguity | Exclude | Because it is not a clearly defined or measurable outcome. |
| 233 | Traumatic events | Include |  |
| 234 | Unprofessional behavior | Include |  |
| 235 | Variety | Include |  |
| 236 | Violence | Exclude | Only partially covered and not a distinct outcome. |
| 237 | Vulnerability to Disease | Exclude | Not considered a direct outcome. |
| 238 | Well-being | Exclude | It is too broad and overarching to function as a discrete outcome. |
| 239 | Wisdom | Include |  |
| 240 | Work ability | Include |  |
| 241 | Work attitude, values, and engagement | Exclude | engagement is already included, rest are not outcomes |
| 242 | Work effectiveness | Exclude | reflects performance, not wellbeing |
| 243 | Work environment | Exclude | already respresented in other constructs |
| 244 | Work excitement | Include |  |
| 245 | Work frustration | Include |  |
| 246 | Work interruption | Include |  |
| 247 | Work life balance | Include |  |
| 248 | Work motivation factors | Exclude | It describes behavioral determinants, not outcomes. |
| 249 | Work values | Include | effect modifier |
| 250 | Working experience | Exclude | Already covered under "nursing experience" |
| 251 | Working memory capacity | Exclude | Due to vagueness and lack of clarity as a construct, personal resource |
| 252 | Workload | Include | Well-established indicator of job-related strain. |
| 253 | Workplace and personal factors | Exclude | both components are too vague to define clearly. |
| 254 | Worrying | Exclude | already covered under 'psychological distress'. |

## Supplemental Table S4. Initial start-set (n=137), including PERMA+4 mapping

| **Item** | **Domain** |
| --- | --- |
| Absenteeism | Accomplishment |
| Building satisfaction | Accomplishment |
| Caring efficacy | Accomplishment |
| Flourishing | Accomplishment |
| Perceived ability to work | Accomplishment |
| Perceived balance between effort and reward | Accomplishment |
| Perceived professional growth | Accomplishment |
| Professional fulfilment | Accomplishment |
| Satisfaction with career identity | Accomplishment |
| Satisfaction with performance at work | Accomplishment |
| Self-efficacy | Accomplishment |
| Wisdom | Accomplishment |
| Fairness of compensation relative to workload | Economic Security |
| Income satisfaction | Economic Security |
| Rewarding | Economic Security |
| Satisfaction with compensation model | Economic Security |
| Altruism | Engagement |
| Autonomy | Engagement |
| Flow | Engagement |
| Goal orientation | Engagement |
| Job crafting | Engagement |
| Participation in decision making | Engagement |
| Perceived quality of leadership | Engagement |
| Perceived support from colleagues | Engagement |
| Professional commitment | Engagement |
| Variety | Engagement |
| Work engagement | Engagement |
| Work life balance | Engagement |
| Calling | Meaning |
| Compassion fatigue | Meaning |
| Experienced gratitude | Meaning |
| Loneliness | Meaning |
| Meaning at work | Meaning |
| Professional values | Meaning |
| Assertiveness | Mindset |
| Cognitive overload at work | Mindset |
| Coping | Mindset |
| Cynicism | Mindset |
| Death-related anxiety | Mindset |
| Difficulty in emotion regulation | Mindset |
| Emotional Intelligence | Mindset |
| Enthusiasm | Mindset |
| Feeling of control | Mindset |
| Feeling prepared | Mindset |
| Grit | Mindset |
| Intrusive memories | Mindset |
| Mental health | Mindset |
| Mindfulness | Mindset |
| Moral distress | Mindset |
| Moral resilience | Mindset |
| Need for recovery | Mindset |
| Neglect of work | Mindset |
| Personality traits | Mindset |
| Professional identity | Mindset |
| Psychological flexibility | Mindset |
| Resilience | Mindset |
| Rumination | Mindset |
| Self-compassion | Mindset |
| Self-confidence in professional role | Mindset |
| Work excitement | Mindset |
| Work values | Mindset |
| Work-related regret | Mindset |
| Fatigue | Physical Health |
| Functional status | Physical Health |
| Pain | Physical Health |
| Perceived mental health-related impairment | Physical Health |
| Presenteeism | Physical Health |
| PTSD | Physical Health |
| Quality of life | Physical Health |
| Self-care | Physical Health |
| Self-rated health | Physical Health |
| Sleep | Physical Health |
| Somatic complaints | Physical Health |
| Work ability | Physical Health |
| Work-related energy | Physical Health |
| Aggressiveness | Positive Emotions |
| Alexithymia | Positive Emotions |
| Anxiety | Positive Emotions |
| Avoidance behaviour | Positive Emotions |
| Burnout | Positive Emotions |
| Depersonalisation | Positive Emotions |
| Depression | Positive Emotions |
| Emotional exhaustion | Positive Emotions |
| Fear | Positive Emotions |
| Grief | Positive Emotions |
| Happiness | Positive Emotions |
| Hope | Positive Emotions |
| Intention to leave | Positive Emotions |
| Job insecurity | Positive Emotions |
| Job satisfaction | Positive Emotions |
| Mood | Positive Emotions |
| Motivation | Positive Emotions |
| Occupational disidentification | Positive Emotions |
| Optimism | Positive Emotions |
| Perceived professional inadequacy | Positive Emotions |
| Previous traumatic work experiences | Positive Emotions |
| Psychological distress | Positive Emotions |
| Psychological Need Satisfaction | Positive Emotions |
| Self-esteem | Positive Emotions |
| Spiritual wellbeing | Positive Emotions |
| Stress | Positive Emotions |
| Traumatic events | Positive Emotions |
| Belongingness | Relationships |
| Bullying | Relationships |
| Conflict | Relationships |
| Experienced collaboration with colleagues | Relationships |
| Hcp-patient relationship | Relationships |
| Interpersonal behaviour | Relationships |
| Intimidation | Relationships |
| Mentorship | Relationships |
| Sense of coherence | Relationships |
| Social connectedness at work | Relationships |
| Social functioning | Relationships |
| Team vitality | Relationships |
| Unprofessional behaviour | Relationships |
| Bureaucracy related stress | Work Environment |
| Climate | Work Environment |
| Work culture | Work Environment |
| Distress from workplace harassment | Work Environment |
| Distress related to aggressive or disruptive behaviours | Work Environment |
| Documentation burden | Work Environment |
| Job control | Work Environment |
| Job fit | Work Environment |
| Job rotation | Work Environment |
| Lifelong learning | Work Environment |
| Nursing governance | Work Environment |
| Organizational constraints | Work Environment |
| Patient-to-HCP ratio | Work Environment |
| Perceived fairness in workplace | Work Environment |
| Perceived organizational support | Work Environment |
| Perceptions of organizational politics | Work Environment |
| Procedural justice | Work Environment |
| Satisfaction with organizational communication style | Work Environment |
| Satisfaction with supervisor | Work Environment |
| Work frustration | Work Environment |
| Work interruption | Work Environment |
| Workload | Work Environment |

## Supplemental Table S5. Composition of panel members across professional roles and geo-economic regions in round 3

| **Professional role** | **High geo-economic** | **Middle geo-eonomic** | **Low geo-economic** | **Total** |
| --- | --- | --- | --- | --- |
| **Round 1** |  |  |  |  |
| Physician | 11 | 8 | 0 | 19 |
| Nurse | 7 | 1 | 6 | 14 |
| Researcher | 10 | 2 | 2 | 14 |
| Allied HCP | 3 | 1 | 6 | 10 |
| Total | 31 | 12 | 14 | 57 |
| **Round 2** |  |  |  |  |
| Physician | 9 | 6 | 0 | 15 |
| Nurse | 7 | 1 | 6 | 14 |
| Researcher | 8 | 2 | 2 | 12 |
| Allied HCP | 3 | 1 | 5 | 9 |
| Total | 27 | 10 | 13 | 50 |
| **Round 3** |  |  |  |  |
| Physician | 9 | 5 | 0 | 14 |
| Nurse | 6 | 1 | 6 | 13 |
| Researcher | 6 | 2 | 2 | 10 |
| Allied HCP | 3 | 1 | 5 | 9 |
| Total | 24 | 9 | 13 | 46 |

## Supplemental Table S6. Results per item from Delphi round 1

| **item** | **Decision** | **%7-9** | **% 1-3** | **median** | **IQR** |
| --- | --- | --- | --- | --- | --- |
| Climate | Inclusion | 96.5% | 0.0% | 8 | 2 |
| Motivation | Inclusion | 96.5% | 0.0% | 8 | 2 |
| Perceived fulfilment | Inclusion | 94.7% | 0.0% | 8 | 2 |
| Work related energy | Inclusion | 94.7% | 0.0% | 8 | 2 |
| Work life balance | Inclusion | 93.0% | 0.0% | 9 | 1 |
| Fairness of compensation relative to workload | Inclusion | 91.2% | 0.0% | 9 | 1 |
| Income satisfaction | Inclusion | 91.2% | 0.0% | 9 | 1 |
| Job satisfaction | Inclusion | 91.2% | 0.0% | 9 | 1 |
| Meaning at work | Inclusion | 91.2% | 0.0% | 8 | 2 |
| Quality of life | Inclusion | 91.2% | 0.0% | 8 | 1 |
| Self-efficacy | Inclusion | 91.2% | 0.0% | 8 | 2 |
| Work ability | Inclusion | 91.2% | 0.0% | 8 | 2 |
| Work culture | Inclusion | 91.2% | 0.0% | 8 | 2 |
| Perceived fairness in workplace | Inclusion | 91.2% | 1.8% | 8 | 2 |
| Belongingness | Inclusion | 89.5% | 0.0% | 8 | 2 |
| Resilience | Inclusion | 89.5% | 0.0% | 8 | 2 |
| Perceived professional growth | Inclusion | 89.5% | 0.0% | 8 | 2 |
| Satisfaction with career identity | Inclusion | 89.5% | 0.0% | 8 | 2 |
| Rewarding | Inclusion | 89.5% | 1.8% | 8 | 2 |
| Participation in decision making | Inclusion | 89.5% | 1.8% | 8 | 2 |
| Team vitality | Inclusion | 89.5% | 1.8% | 8 | 2 |
| Burnout | Inclusion | 89.5% | 8.8% | 9 | 1 |
| Sleep | Inclusion | 87.7% | 0.0% | 8 | 2 |
| Mental health | Inclusion | 87.7% | 0.0% | 8 | 1 |
| Perceived balance between effort and reward | Inclusion | 87.7% | 0.0% | 8 | 2 |
| Satisfaction with performance at work | Inclusion | 87.7% | 0.0% | 8 | 2 |
| Work values | Inclusion | 87.7% | 3.5% | 9 | 2 |
| Coping | Inclusion | 86.0% | 0.0% | 8 | 1 |
| Experienced collaboration with colleagues | Inclusion | 86.0% | 0.0% | 8 | 2 |
| Self confidence in professional role | Inclusion | 86.0% | 3.5% | 8 | 2 |
| Satisfaction with compensation model | No consensus | 84.2% | 0.0% | 8 | 2 |
| Work engagement | No consensus | 84.2% | 0.0% | 8 | 2 |
| Need for recovery | No consensus | 84.2% | 1.8% | 8 | 2 |
| Perceived organizational support | No consensus | 84.2% | 1.8% | 8 | 2 |
| Perceived support from colleagues | No consensus | 84.2% | 3.5% | 8 | 2 |
| Job fit | No consensus | 82.5% | 0.0% | 8 | 1 |
| Perceived ability to work | No consensus | 82.5% | 0.0% | 8 | 2 |
| Professional commitment | No consensus | 82.5% | 1.8% | 8 | 2 |
| Self-care | No consensus | 82.5% | 1.8% | 8 | 2 |
| Healthcare professional patient relationship | No consensus | 82.5% | 3.5% | 8 | 2 |
| Perceived mental health related impairment | No consensus | 82.5% | 3.5% | 8 | 2 |
| Perceived quality of leadership | No consensus | 82.5% | 3.5% | 8 | 2 |
| Patient to hcp ratio | No consensus | 82.5% | 5.3% | 8 | 2 |
| Distress from workplace harassment | No consensus | 82.5% | 10.5% | 8 | 2 |
| Caring efficacy | No consensus | 80.7% | 0.0% | 7,5 | 1 |
| Functional status | No consensus | 80.7% | 0.0% | 8 | 2 |
| Professional values | No consensus | 80.7% | 0.0% | 9 | 2 |
| Psychological flexibility | No consensus | 80.7% | 1.8% | 8 | 2 |
| Satisfaction with supervisor | No consensus | 80.7% | 1.8% | 8 | 2 |
| Social connectedness at work | No consensus | 80.7% | 1.8% | 7 | 2 |
| Fatigue | No consensus | 80.7% | 5.3% | 8 | 2 |
| Psychological distress | No consensus | 80.7% | 7.0% | 8 | 2 |
| Anxiety | No consensus | 80.7% | 8.8% | 8 | 2 |
| Emotional exhaustion | No consensus | 80.7% | 8.8% | 8 | 2 |
| Distress related to aggressive or disruptive behaviour | No consensus | 80.7% | 10.5% | 8 | 2 |
| Professional identity | No consensus | 78.9% | 0.0% | 8 | 2 |
| Autonomy | No consensus | 78.9% | 1.8% | 8 | 2 |
| Happiness | No consensus | 78.9% | 3.5% | 8 | 2 |
| Lifelong learning | No consensus | 78.9% | 3.5% | 8 | 2 |
| Emotional intelligence | No consensus | 78.9% | 5.3% | 8 | 2 |
| Depression | No consensus | 78.9% | 8.8% | 8 | 2 |
| Work frustration | No consensus | 78.9% | 8.8% | 8 | 2 |
| Exposure to aggressiveness | No consensus | 78.9% | 12.3% | 8 | 2 |
| Mentorship | No consensus | 77.2% | 0.0% | 8 | 2 |
| Experienced gratitude | No consensus | 77.2% | 0.0% | 8 | 2 |
| Work excitement | No consensus | 77.2% | 0.0% | 8 | 2 |
| Interpersonal behaviour | No consensus | 77.2% | 1.8% | 7 | 2 |
| Cognitive overload at work | No consensus | 77.2% | 3.5% | 8 | 2 |
| Intimidation | No consensus | 77.2% | 7.0% | 8 | 2 |
| Bullying | No consensus | 77.2% | 8.8% | 8 | 2 |
| Workload | No consensus | 77.2% | 10.5% | 8 | 2 |
| Enthusiasm | No consensus | 75.4% | 0.0% | 8 | 1 |
| Feeling prepared | No consensus | 75.4% | 0.0% | 8 | 2 |
| Psychological need satisfaction | No consensus | 75.4% | 1.8% | 8 | 2 |
| Stress | No consensus | 75.4% | 3.5% | 8 | 2 |
| Sense of coherence | No consensus | 75.4% | 3.5% | 7 | 2 |
| Conflict at work | No consensus | 75.4% | 7.0% | 8 | 2 |
| Unprofessional behaviour | No consensus | 75.4% | 12.3% | 8 | 2 |
| Wisdom | No consensus | 73.7% | 5.3% | 8 | 3 |
| PTSD | No consensus | 73.7% | 8.8% | 8 | 2 |
| Job insecurity | No consensus | 73.7% | 12.3% | 8 | 2 |
| Perceived professional inadequacy | No consensus | 73.7% | 12.3% | 8 | 2,25 |
| Flourishing | No consensus | 71.9% | 1.8% | 7 | 2 |
| Goal orientation | No consensus | 71.9% | 1.8% | 7 | 3 |
| Mood | No consensus | 71.9% | 3.5% | 8 | 3 |
| Moral resilience | No consensus | 71.9% | 3.5% | 8 | 3 |
| Self esteem | No consensus | 71.9% | 5.3% | 8 | 3 |
| Feeling in control | No consensus | 70.2% | 1.8% | 8 | 3 |
| Job control | No consensus | 70.2% | 1.8% | 8 | 3 |
| Self-compassion | No consensus | 70.2% | 1.8% | 8 | 3 |
| Social functioning | No consensus | 70.2% | 1.8% | 7 | 3 |
| Pain | No consensus | 70.2% | 7.0% | 7 | 3 |
| Loneliness | No consensus | 70.2% | 14.0% | 8 | 3 |
| Assertiveness | No consensus | 68.4% | 1.8% | 8 | 3 |
| Hope | No consensus | 68.4% | 1.8% | 8 | 3 |
| Mindfulness | No consensus | 68.4% | 1.8% | 7 | 2,25 |
| Self-rated health | No consensus | 68.4% | 1.8% | 7 | 3 |
| Optimism | No consensus | 68.4% | 5.3% | 8 | 3 |
| Procedural justice | No consensus | 68.4% | 5.3% | 8 | 2 |
| Avoidance behaviour | No consensus | 68.4% | 7.0% | 7 | 3 |
| Compassion fatigue | No consensus | 68.4% | 7.0% | 7,5 | 2,25 |
| Job crafting | No consensus | 66.7% | 1.8% | 7 | 3 |
| Documentation burden | No consensus | 66.7% | 3.5% | 7 | 2 |
| Fear | No consensus | 66.7% | 5.3% | 7 | 2,25 |
| Moral distress | No consensus | 66.7% | 5.3% | 7 | 2 |
| Organizational constraints | No consensus | 66.7% | 5.3% | 7 | 3 |
| Somatic complaints at work | No consensus | 66.7% | 5.3% | 7 | 2 |
| Depersonalisation | No consensus | 66.7% | 8.8% | 8 | 3 |
| Previous traumatic work experiences | No consensus | 66.7% | 8.8% | 8 | 3 |
| Traumatic events | No consensus | 66.7% | 10.5% | 8 | 2 |
| Satisfaction with organizational communication style | No consensus | 64.9% | 0.0% | 8 | 3 |
| Calling | No consensus | 64.9% | 1.8% | 7 | 2,5 |
| Perceptions of organizational politics | No consensus | 64.9% | 1.8% | 7 | 2 |
| Bureaucracy related stress | No consensus | 64.9% | 7.0% | 7 | 2 |
| Occupational disidentification | No consensus | 64.9% | 14.0% | 8 | 2 |
| Presenteeism | No consensus | 63.2% | 1.8% | 8 | 3 |
| Building satisfaction | No consensus | 63.2% | 3.5% | 7 | 3 |
| Nursing governance | No consensus | 63.2% | 3.5% | 7,5 | 3 |
| Intention to leave | No consensus | 63.2% | 10.5% | 8 | 3 |
| Flow | No consensus | 61.4% | 0.0% | 7 | 3 |
| Variety | No consensus | 61.4% | 3.5% | 7 | 2 |
| Work interruption | No consensus | 59.6% | 12.3% | 7 | 2 |
| Rumination | No consensus | 57.9% | 3.5% | 7 | 3 |
| Personal traits | No consensus | 57.9% | 10.5% | 7 | 2 |
| Difficulty in emotion regulation | No consensus | 57.9% | 12.3% | 7 | 2 |
| Neglect of work | No consensus | 57.9% | 14.0% | 7 | 4 |
| Job rotation | No consensus | 56.1% | 5.3% | 7 | 2 |
| Work related regret | No consensus | 56.1% | 14.0% | 7 | 2,25 |
| Spiritual well being | No consensus | 54.4% | 10.5% | 7 | 4 |
| Absenteeism | No consensus | 54.4% | 12.3% | 7 | 3 |
| Grit | No consensus | 52.6% | 3.5% | 7 | 3 |
| Grief | No consensus | 52.6% | 8.8% | 7 | 2 |
| Altruism | No consensus | 50.9% | 7.0% | 7 | 3 |
| Intrusive memories | No consensus | 50.9% | 8.8% | 7 | 3 |
| Alexithymia | Exclusion | 45.6% | 12.3% | 7 | 3 |
| Cynicism | Exclusion | 43.9% | 14.0% | 6 | 4 |
| Death related anxiety | Exclusion | 40.4% | 17.5% | 6 | 3 |

## Supplemental Table S7. Suggestions from round 1 and actions on it

|  | **Suggestion** | **Response/action** |
| --- | --- | --- |
| 1 | Assessment of physical activity | No outcome |
| 2 | I feel that all listed items, in a broader sense, are related to the wellbeing of healthcare workers. I think that mental health factors are significant, for instance, including their level of depersonalization and emotional exhaustion at work. | Comment, no suggestion for new outcome |
| 3 | not just working hour is important but flexible duty roster family time matters | Clustered to “family wellbeing” |
| 4 | CPD(continues professional development ) | Falls underneath “perceived professional growth” |
| 5 | onboarding | No outcome |
| 6 | Leave related questionnaires for wellbeing for eg- forced leave system in bank | Falls underneath “intention to leave” |
| 7 | Social environment at home | Clustered to “family wellbeing” |
| 8 | The health system shall work on the development of non-physician clinician in different decision making and to have clear career path that extend to the date latest competency, like that of the medicine program. | Falls underneath “perceived professional growth” |
| 9 | National Peace | External |
| 10 | Psychological and Emotional Well-Being | Captured in multiple constructs in survey already |
| 11 | Cultural well being | Vague, unclear suggestion |
| 12 | Incentives every health professional |  |
| 13 | Giving more attention for healthcare professionals, if possible, give more time to relax. | Not an outcome |
| 14 | Patient to Nurse Relation | Falls underneath “patient to HCP ratio” |
| 15 | Ease of access to healthcare | Not an outcome relevant to HCP |
| 16 | Supported rota team, annual and study leaves when required | Falls underneath “perceived organizational support” |
| 17 | I think it is important to distinguish seniority in career. For example, a junior doctor is probably less affected by lack of autonomy in their work when compared to a senior physician | No suggestion/outcome |
| 18 | Conciliation of family and work life. Work-Family conciliation strategies | Clustered to “family wellbeing” |
| 19 | I think that family issues is an important component to keep in mind. | Clustered to “family wellbeing” |
| 20 | Trust in immediate leader | Falls underneath “perceived quality of leadership” |
| 21 | sexual harassment | Falls underneath “harassment” |
| 22 | Sleep | Already in questionnaire |
| 23 | Moral Distress and Ethical Stress | “Moral distress” is already in the questionnaire |
| 24 | Family wellbeing | Clustered to “family wellbeing” |
| 25 | Variety in work/ amount of tasks | Falls underneath “workload” and “variety” |
| 26 | timely salary and insurance benefit increases support to individual staff | Falls underneath “satisfaction with compensation model” |
| 27 | Shifts | Clustered to “irregular working” |
| 28 | The health system shall stop consider non-physician as a subordinate. The current health system looks like a feudal system where few selected entitled for the privilege. | Clustered to “professional equality” |
| 29 | Patient Satisfaction | Not an HCP wellbeing related outcome |
| 30 | Workload and Work Environment | Already in the survey |
| 31 | Ethical dilemma | Falls underneath “moral distress” |
| 32 | Respect by others | Clustered to “respect” |
| 33 | Communication | Already in survey |
| 34 | Well-being of family members | Clustered to “family wellbeing” |
| 35 | Study budget to cover cost of professional exams and courses | Falls underneath lifelong learning/ professional growth |
| 36 | Health System sustainability (to maintain value in the face of increasing demand and cost) | Clustered to “sustainability” |
| 37 | Trust in organization leader | Falls underneath “perceived quality of leadership” |
| 38 | cross-border activities | Unclear outcome/suggestion |
| 39 | Perceived Organizational Support | Already in survey |
| 40 | recognition of moral support and effort reduces stress | Falls underneath moral distress and support. |
| 41 | Payment shall improve, especially in a country like Ethiopia, where the highest payment of specialized doctor is less than 150USD/month. | Falls underneath satisfaction with compensation model. |
| 42 | Access to Medical Equipment | Clustered to “equipment” |
| 43 | Economic and Social Security | Already in survey |
| 44 | Interprofessional integration | Falls underneath Social connectedness at work |
| 45 | Commute to work | Falls underneath work-life balance |
| 46 | Improved Staffing out of hours | Falls underneath workload or patient to HCP ratio |
| 47 | Societal support for job role/mission | Already in survey |
| 48 | inappropriate behaviour | Already in survey |
| 49 | Work-Life Integration Satisfaction | Falls underneath work-life balance |
| 50 | providing equal opportunities and career progression enhances personal satisfaction | Clustered to “professional equality” |
| 51 | Professional Growth and Motivation | Already in survey |
| 52 | Keep the mental wellbeing of health professionals | No outcome |
| 53 | Physical safety/assault | Falls underneath workplace harassment |
| 54 | Psychological Safety in the Workplace | Clustered to “Save environment” |

## Supplemental Table S8. Results per item from Delphi round 2

| **Item** | **Decision** | **% 7-9** | **%1-3** | **median** | **IQR** |
| --- | --- | --- | --- | --- | --- |
| Fatigue | Inclusion | 96.0% | 0.0% | 8 | 1 |
| Perceived organizational support | Inclusion | 96.0% | 0.0% | 8 | 2 |
| Satisfaction with compensation model | Inclusion | 96.0% | 0.0% | 8 | 1 |
| Professional values | Inclusion | 92.0% | 0.0% | 8 | 1,75 |
| Functional status | Inclusion | 92.0% | 2.0% | 8 | 1,75 |
| Patient to hcp ratio | Inclusion | 92.0% | 2.0% | 8 | 2 |
| Perceived quality of leadership | Inclusion | 90.0% | 0.0% | 8 | 2 |
| Perceived support from colleagues | Inclusion | 90.0% | 0.0% | 8 | 1,75 |
| Satisfaction with supervisor | Inclusion | 90.0% | 0.0% | 8 | 2 |
| Social connectedness at work | Inclusion | 90.0% | 0.0% | 8 | 1 |
| Job insecurity | Inclusion | 90.0% | 2.0% | 8 | 1,75 |
| Professional commitment | Inclusion | 90.0% | 2.0% | 8 | 1 |
| Professional identity | Inclusion | 90.0% | 2.0% | 8 | 1 |
| Work engagement | Inclusion | 90.0% | 2.0% | 8 | 1,75 |
| Psychological distress | Inclusion | 90.0% | 4.0% | 8 | 1 |
| Mentorship | Inclusion | 88.0% | 0.0% | 8 | 2 |
| Psychological flexibility | Inclusion | 88.0% | 0.0% | 8 | 1 |
| Self-care | Inclusion | 88.0% | 0.0% | 8 | 1 |
| Anxiety | Inclusion | 88.0% | 2.0% | 8 | 1 |
| Enthusiasm | Inclusion | 88.0% | 2.0% | 8 | 1 |
| Stress | Inclusion | 88.0% | 2.0% | 8 | 1 |
| Professional equality | Inclusion | 88.0% | 2.0% | 8 | 2 |
| Depression | Inclusion | 88.0% | 4.0% | 8 | 1 |
| Procedural justice | Inclusion | 86.0% | 0.0% | 8 | 1 |
| Happiness | Inclusion | 86.0% | 2.0% | 8 | 2 |
| Emotional intelligence | Inclusion | 86.0% | 2.0% | 8 | 1,75 |
| Interpersonal behaviour | Inclusion | 86.0% | 2.0% | 8 | 1 |
| Job fit | Inclusion | 86.0% | 2.0% | 8 | 1 |
| Work excitement | Inclusion | 86.0% | 2.0% | 8 | 1 |
| Need for recovery | Inclusion | 86.0% | 4.0% | 8 | 1 |
| Previous traumatic work experiences | Inclusion | 86.0% | 4.0% | 7 | 1 |
| Cognitive overload at work | Inclusion | 86.0% | 6.0% | 8 | 1 |
| Experienced gratitude | No consensus | 84.0% | 0.0% | 8 | 1 |
| Self esteem | No consensus | 84.0% | 0.0% | 8 | 1 |
| Autonomy | No consensus | 84.0% | 2.0% | 8 | 1 |
| Self-compassion | No consensus | 84.0% | 2.0% | 8 | 1 |
| Distress from workplace harassment | No consensus | 84.0% | 4.0% | 8 | 2 |
| Conflict at work | No consensus | 84.0% | 6.0% | 8 | 2 |
| Emotional exhaustion | No consensus | 84.0% | 6.0% | 8 | 2 |
| Unprofessional behaviour | No consensus | 84.0% | 8.0% | 8 | 1 |
| Feeling prepared | No consensus | 82.0% | 0.0% | 8 | 1,75 |
| Self-rated health | No consensus | 82.0% | 0.0% | 8 | 1 |
| Sense of coherence | No consensus | 82.0% | 0.0% | 7 | 1 |
| Compassion fatigue | No consensus | 82.0% | 2.0% | 7 | 1 |
| Healthcare professional patient relationship | No consensus | 82.0% | 2.0% | 8 | 2 |
| Perceived professional inadequacy | No consensus | 82.0% | 2.0% | 7 | 1 |
| Depersonalisation | No consensus | 82.0% | 4.0% | 7 | 1 |
| Workload | No consensus | 82.0% | 4.0% | 8 | 2 |
| Caring efficacy | No consensus | 82.0% | 4.0% | 7 | 1 |
| Neglect of work | No consensus | 82.0% | 8.0% | 7 | 1 |
| Feeling in control | No consensus | 80.0% | 0.0% | 8 | 1 |
| Lifelong learning | No consensus | 80.0% | 2.0% | 8 | 2 |
| Moral distress | No consensus | 80.0% | 2.0% | 7 | 1 |
| Intimidation | No consensus | 80.0% | 4.0% | 7 | 1 |
| Perceived mental health related impairment | No consensus | 80.0% | 4.0% | 8 | 1 |
| Work frustration | No consensus | 80.0% | 8.0% | 8 | 1,75 |
| Job control | No consensus | 78.0% | 0.0% | 7,5 | 1 |
| Flourishing | No consensus | 78.0% | 2.0% | 7 | 1 |
| Perceived ability to work | No consensus | 78.0% | 2.0% | 7 | 1 |
| Psychological need satisfaction | No consensus | 78.0% | 2.0% | 7 | 1 |
| Hope | No consensus | 78.0% | 4.0% | 7,5 | 1 |
| Personal traits | No consensus | 78.0% | 4.0% | 7 | 1 |
| Family wellbeing | No consensus | 76.0% | 0.0% | 8 | 2 |
| Goal orientation | No consensus | 76.0% | 2.0% | 7 | 1 |
| Organizational constraints | No consensus | 76.0% | 2.0% | 7 | 1 |
| Somatic complaints at work | No consensus | 76.0% | 4.0% | 7 | 1 |
| Bullying | No consensus | 76.0% | 6.0% | 8 | 2 |
| Job crafting | No consensus | 74.0% | 0.0% | 7 | 1 |
| Presenteeism | No consensus | 74.0% | 2.0% | 7 | 1,75 |
| Intention to leave | No consensus | 74.0% | 4.0% | 8 | 2,75 |
| Occupational disidentification | No consensus | 74.0% | 4.0% | 7,5 | 1,75 |
| Optimism | No consensus | 72.0% | 0.0% | 8 | 2 |
| Social functioning | No consensus | 72.0% | 0.0% | 7 | 1,75 |
| Wisdom | No consensus | 72.0% | 2.0% | 7,5 | 2 |
| Exposure to aggressiveness | No consensus | 72.0% | 4.0% | 8 | 3 |
| Pain | No consensus | 72.0% | 6.0% | 7 | 2 |
| Traumatic events | No consensus | 72.0% | 6.0% | 7 | 2 |
| Flow | No consensus | 70.0% | 0.0% | 7 | 1,75 |
| Avoidance behaviour | No consensus | 70.0% | 0.0% | 7 | 2 |
| Building satisfaction | No consensus | 70.0% | 0.0% | 7 | 2 |
| Moral resilience | No consensus | 70.0% | 2.0% | 7 | 1,75 |
| Perceptions of organizational politics | No consensus | 70.0% | 2.0% | 7 | 2 |
| PTSD | No consensus | 70.0% | 4.0% | 7,5 | 2 |
| Work related regret | No consensus | 70.0% | 4.0% | 7 | 1,75 |
| Variety | No consensus | 68.0% | 2.0% | 7 | 2 |
| Bureaucracy related stress | No consensus | 68.0% | 2.0% | 7 | 2 |
| Rumination | No consensus | 68.0% | 4.0% | 7 | 2 |
| Difficulty in emotion regulation | No consensus | 68.0% | 4.0% | 7 | 1 |
| Assertiveness | No consensus | 66.0% | 2.0% | 8 | 2 |
| Mood | No consensus | 66.0% | 4.0% | 7 | 2 |
| Mindfulness | No consensus | 66.0% | 6.0% | 7 | 2 |
| Fear | No consensus | 64.0% | 0.0% | 7 | 2 |
| Nursing governance | No consensus | 64.0% | 6.0% | 7 | 2 |
| Grit | No consensus | 62.0% | 4.0% | 7 | 2 |
| Loneliness | No consensus | 62.0% | 4.0% | 7 | 2 |
| Documentation burden | No consensus | 62.0% | 4.0% | 7 | 2 |
| Work interruption | No consensus | 60.0% | 4.0% | 7 | 2 |
| Calling | No consensus | 58.0% | 4.0% | 7 | 2 |
| Absenteeism | No consensus | 58.0% | 8.0% | 7 | 2 |
| Spiritual well being | No consensus | 58.0% | 8.0% | 7 | 3 |
| Altruism | No consensus | 56.0% | 2.0% | 7 | 2 |
| Intrusive memories | No consensus | 54.0% | 4.0% | 7 | 1 |
| Grief | No consensus | 54.0% | 6.0% | 7 | 2 |
| Job rotation | Exclusion | 48.0% | 4.0% | 6,5 | 2 |
| Distress related to aggressive or disruptive behaviour | Exclusion | 32.0% | 0.0% | 8,5 | 2 |
| Satisfaction with organizational communication style | Exclusion | 26.0% | 0.0% | 7,5 | 1 |

## Supplemental Table S9. Results from Delphi round 3, all panel members

| **Construct** | **Times ranked in top 10** | **% ranked in top 10** |
| --- | --- | --- |
| Work life balance | 35 | 76,1 |
| Job satisfaction | 28 | 60,9 |
| Quality of life | 22 | 47,8 |
| Motivation | 21 | 45,7 |
| Mental health | 21 | 45,7 |
| Income satisfaction | 19 | 41,3 |
| Meaning at work | 18 | 39,1 |
| Perceived professional growth | 15 | 32,6 |
| Work culture | 14 | 30,4 |
| Perceived fulfilment | 12 | 26,1 |
| Burnout | 12 | 26,1 |
| Stress | 10 | 21,7 |
| Satisfaction with career identity | 9 | 19,6 |
| Participation in decision making | 9 | 19,6 |
| Work related energy | 8 | 17,4 |
| Sleep | 8 | 17,4 |
| Perceived organizational support | 8 | 17,4 |
| Job insecurity | 8 | 17,4 |
| Work engagement | 8 | 17,4 |
| Fairness of compensation relative to workload | 7 | 15,2 |
| Perceived fairness in workplace | 7 | 15,2 |
| Belongingness | 7 | 15,2 |
| Resilience | 7 | 15,2 |
| Experienced collaboration with colleagues | 7 | 15,2 |
| Perceived support from colleagues | 7 | 15,2 |
| Self care | 7 | 15,2 |
| Happiness | 7 | 15,2 |
| Emotional intelligence | 7 | 15,2 |
| Perceived balance between effort and reward | 6 | 13 |
| Professional values | 6 | 13 |
| Patient-HCP-ratio | 6 | 13 |
| Satisfaction with supervisor | 6 | 13 |
| Job fit | 6 | 13 |
| Climate | 5 | 10,9 |
| Work values | 5 | 10,9 |
| Psychological distress | 5 | 10,9 |
| Interpersonal behaviour | 5 | 10,9 |
| Self efficacy | 4 | 8,7 |
| Satisfaction with performance at work | 4 | 8,7 |
| Self confidence in professional role | 4 | 8,7 |
| Fatigue | 4 | 8,7 |
| Perceived quality of leadership | 4 | 8,7 |
| Professional identity | 4 | 8,7 |
| Cognitive overload at work | 4 | 8,7 |
| Team vitality | 3 | 6,5 |
| Coping | 3 | 6,5 |
| Social connectedness at work | 3 | 6,5 |
| Professional commitment | 3 | 6,5 |
| Mentorship | 3 | 6,5 |
| Professional equality | 3 | 6,5 |
| Work excitement | 3 | 6,5 |
| Need for recovery | 3 | 6,5 |
| Rewarding | 2 | 4,3 |
| Satisfaction with compensation model | 2 | 4,3 |
| Functional status | 2 | 4,3 |
| Psychological flexibility | 1 | 2,2 |
| Enthusiasm | 1 | 2,2 |
| Depression | 1 | 2,2 |
| Previous traumatic work experiences | 1 | 2,2 |
| Work ability | 0 | 0 |
| Anxiety | 0 | 0 |
| Procedural justice | 0 | 0 |

## Supplemental Table S10. Results from Delphi round 3, clinical panel members

| **Construct** | **Times ranked in top 10** | **% ranked in top 10** |
| --- | --- | --- |
| Work life balance | 30 | 83,3 |
| Job satisfaction | 24 | 66,7 |
| Quality of life | 19 | 52,8 |
| Motivation | 16 | 44,4 |
| Mental health | 16 | 44,4 |
| Meaning at work | 14 | 38,9 |
| Income satisfaction | 13 | 36,1 |
| Work culture | 12 | 33,3 |
| Perceived professional growth | 10 | 27,8 |
| Stresswork | 9 | 25 |
| Satisfaction with career identity | 8 | 22,2 |
| Participation in decision making | 8 | 22,2 |
| Burnout | 8 | 22,2 |
| Perceived fulfilment | 7 | 19,4 |
| Work related energy | 7 | 19,4 |
| Belongingness | 7 | 19,4 |
| Sleep | 7 | 19,4 |
| Job insecurity | 7 | 19,4 |
| Perceived fairness in workplace | 6 | 16,7 |
| Perceived organizational support | 6 | 16,7 |
| Perceived support from colleagues | 6 | 16,7 |
| Work engagement | 6 | 16,7 |
| Self care | 6 | 16,7 |
| Happiness | 6 | 16,7 |
| Job fit | 6 | 16,7 |
| Fairness of compensation relative to workload | 5 | 13,9 |
| Work values | 5 | 13,9 |
| Experienced collaboration with colleagues | 5 | 13,9 |
| Psychological distress | 5 | 13,9 |
| Resilience | 4 | 11,1 |
| Satisfaction with performance at work | 4 | 11,1 |
| Perceived quality of leadership | 4 | 11,1 |
| Satisfaction with supervisor | 4 | 11,1 |
| Emotional intelligence | 4 | 11,1 |
| Interpersonal behaviour | 4 | 11,1 |
| Climate | 3 | 8,3 |
| Self efficacy | 3 | 8,3 |
| Team vitality | 3 | 8,3 |
| Perceived balance between effort and reward | 3 | 8,3 |
| Self confidence in professional role | 3 | 8,3 |
| Fatigue | 3 | 8,3 |
| Professional values | 3 | 8,3 |
| Patient-HCP-ratio | 3 | 8,3 |
| Professional identity | 3 | 8,3 |
| Mentorship | 3 | 8,3 |
| Cognitive overload at work | 3 | 8,3 |
| Rewarding | 2 | 5,6 |
| Coping | 2 | 5,6 |
| Satisfaction with compensation model | 2 | 5,6 |
| Social connectedness at work | 2 | 5,6 |
| Professional commitment | 2 | 5,6 |
| Professional equality | 2 | 5,6 |
| Work excitement | 2 | 5,6 |
| Functional status | 1 | 2,8 |
| Psychological flexibility | 1 | 2,8 |
| Depression | 1 | 2,8 |
| Need for recovery | 1 | 2,8 |
| Previous traumatic work experiences | 1 | 2,8 |
| Work ability | 0 | 0 |
| Anxiety | 0 | 0 |
| Enthusiasm | 0 | 0 |
| Procedural justice | 0 | 0 |

## Supplemental Table S11. Weighted ranking of constructs, all panel members

| **Rank** | **Construct** | **Weighted Percentage** |
| --- | --- | --- |
| 1 | Work life balance | 60,1 |
| 2 | Job satisfaction | 48,1 |
| 3 | Mental health | 47,4 |
| 4 | Income satisfaction | 46 |
| 5 | Motivation | 44,5 |
| 6 | Quality of life | 37,2 |
| 7 | Perceived professional growth | 32,5 |
| 8 | Meaning at work | 28,1 |
| 9 | Perceived fulfilment | 28,1 |
| 10 | Work culture | 26,2 |
| 11 | Satisfaction with career identity | 21,4 |
| 12 | Burnout | 20,5 |
| 13 | Patient-HCP-ratio | 18,1 |
| 14 | Participation in decision making | 17,6 |
| 15 | Job insecurity | 16,4 |
| 16 | Climate | 15,3 |
| 17 | Work related energy | 14,8 |
| 18 | Emotional intelligence | 14,2 |
| 19 | Professional values | 14,1 |
| 20 | Sleep | 13,9 |
| 21 | Fairness of compensation relative to workload | 13,4 |
| 22 | Perceived fairness in workplace | 13,2 |
| 23 | Perceived balance between effort and reward | 13 |
| 24 | Happiness | 12,4 |
| 25 | Resilience | 12 |
| 26 | Perceived organizational support | 11,4 |
| 27 | Stress | 11,4 |
| 28 | Experienced collaboration with colleagues | 11,1 |
| 29 | Satisfaction with supervisor | 10,6 |
| 30 | Work engagement | 8,8 |
| 31 | Self care | 8,6 |
| 32 | Job fit | 8,1 |
| 33 | Self efficacy | 7,9 |
| 34 | Interpersonal behaviour | 7,5 |
| 35 | Belongingness | 7,4 |
| 36 | Self confidence in professional role | 7,4 |
| 37 | Professional equality | 7,2 |
| 38 | Team vitality | 6,8 |
| 39 | Perceived support from colleagues | 6,7 |
| 40 | Coping | 6,5 |
| 41 | Perceived quality of leadership | 6,5 |
| 42 | Fatigue | 6,1 |
| 43 | Social connectedness at work | 6 |
| 44 | Professional identity | 5,8 |
| 45 | Functional status | 5,6 |
| 46 | Professional commitment | 5,6 |
| 47 | Psychological distress | 5,4 |
| 48 | Work values | 5,1 |
| 49 | Cognitive overload at work | 4,6 |
| 50 | Work excitement | 4,4 |
| 51 | Enthusiasm | 4,2 |
| 52 | Need for recovery | 3,7 |
| 53 | Satisfaction with performance at work | 3,7 |
| 54 | Rewarding | 3,1 |
| 55 | Mentorship | 2,8 |
| 56 | Previous traumatic work experiences | 1,7 |
| 57 | Psychological flexibility | 1,4 |
| 58 | Depression | 0,9 |
| 59 | Satisfaction with compensation model | 0,9 |
| 60 | Anxiety | 0 |
| 61 | Procedural justice | 0 |
| 62 | Work ability | 0 |

## Supplemental Table S12. Weighted ranking of constructs, clinical panel members

| **Rank** | **Construct** | **Weighted Percentage** |
| --- | --- | --- |
| 1 | Work life balance | 76,7 |
| 2 | Job satisfaction | 58,6 |
| 3 | Motivation | 53,1 |
| 4 | Quality of life | 50,1 |
| 5 | Mental health | 40,4 |
| 6 | Income satisfaction | 36,2 |
| 7 | Work culture | 31,6 |
| 8 | Perceived professional growth | 27,9 |
| 9 | Satisfaction with career identity | 27 |
| 10 | Meaning at work | 25,8 |
| 11 | Participation in decision making | 21,6 |
| 12 | Job fit | 20,9 |
| 13 | Work engagement | 19,1 |
| 14 | Perceived quality of leadership | 17,9 |
| 15 | Work values | 17,9 |
| 16 | Perceived fulfilment | 17,8 |
| 17 | Emotional intelligence | 17 |
| 18 | Resilience | 16,8 |
| 19 | Job insecurity | 15,8 |
| 20 | Climate | 15,6 |
| 21 | Stress | 15,6 |
| 22 | Burnout | 15,1 |
| 23 | Work related energy | 14,9 |
| 24 | Perceived fairness in workplace | 14 |
| 25 | Belongingness | 13,6 |
| 26 | Professional commitment | 13 |
| 27 | Perceived support from colleagues | 12,5 |
| 28 | Self care | 11,9 |
| 29 | Sleep | 11,9 |
| 30 | Happiness | 11 |
| 31 | Perceived organizational support | 11 |
| 32 | Fairness of compensation relative to workload | 9,4 |
| 33 | Psychological distress | 9,4 |
| 34 | Satisfaction with supervisor | 8,6 |
| 35 | Interpersonal behaviour | 8,1 |
| 36 | Patient-HCP-ratio | 8,1 |
| 37 | Experienced collaboration with colleagues | 7,4 |
| 38 | Satisfaction with performance at work | 7,2 |
| 39 | Team vitality | 7,2 |
| 40 | Self efficacy | 6,8 |
| 41 | Professional identity | 5,9 |
| 42 | Fatigue | 5,7 |
| 43 | Professional values | 5,3 |
| 44 | Satisfaction with compensation model | 4,9 |
| 45 | Cognitive overload at work | 4,3 |
| 46 | Perceived balance between effort and reward | 4,3 |
| 47 | Self confidence in professional role | 4,3 |
| 48 | Professional equality | 4,1 |
| 49 | Rewarding | 4,1 |
| 50 | Work excitement | 4,1 |
| 51 | Mentorship | 3,7 |
| 52 | Coping | 3,1 |
| 53 | Social connectedness at work | 2,5 |
| 54 | Previous traumatic work experiences | 2,2 |
| 55 | Functional status | 1,9 |
| 56 | Psychological flexibility | 1,9 |
| 57 | Depression | 1,2 |
| 58 | Need for recovery | 1,2 |
| 59 | Anxiety | 0 |
| 60 | Enthusiasm | 0 |
| 61 | Procedural justice | 0 |
| 62 | Work ability | 0 |

## Supplemental Table S13. Weighted ranking of constructs according to REPS, all panel members

| **Rank** | **Construct** |
| --- | --- |
| 1 | Work life balance |
| 2 | Job satisfaction |
| 3 | Motivation |
| 4 | Mental health |
| 5 | Quality of life |
| 6 | Perceived professional growth |
| 7 | Income satisfaction |
| 8 | Meaning at work |
| 9 | Work culture |
| 10 | Perceived fulfilment |
| 11 | Burnout |
| 12 | Participation in decision making |
| 13 | Work engagement |
| 14 | Stress |
| 15 | Satisfaction with career identity |
| 16 | Sleep |
| 17 | Perceived organizational support |
| 18 | Emotional intelligence |
| 19 | Perceived support from colleagues |
| 20 | Job insecurity |
| 21 | Experienced collaboration with colleagues |
| 22 | Resilience |
| 23 | Happiness |
| 24 | Work related energy |
| 25 | Belongingness |
| 26 | Fairness of compensation relative to workload |
| 27 | Perceived fairness in workplace |
| 28 | Patient-HCP-ratio |
| 29 | Self care |
| 30 | Job fit |
| 31 | Professional values |
| 32 | Satisfaction with supervisor |
| 33 | Perceived balance between effort and reward |
| 34 | Interpersonal behaviour |
| 35 | Work values |
| 36 | Psychological distress |
| 37 | Climate |
| 38 | Self confidence in professional role |
| 39 | Perceived quality of leadership |
| 40 | Self efficacy |
| 41 | Professional identity |
| 42 | Satisfaction with performance at work |
|  | Cognitive overload at work |
| 43 | Fatigue |
| 44 | Mentorship |
| 45 | Coping |
|  | Social connectedness at work |
| 46 | Professional commitment |
|  | Professional equality |
|  | Work excitement |
| 47 | Need for recovery |
| 48 | Team vitality |
| 49 | Rewarding |
|  | Satisfaction with compensation model |
|  | Functional status |
| 50 | Psychological flexibility |
|  | Enthusiasm |
|  | Depression |
|  | Previous traumatic work experiences |
| 51 | Procedural justice |
|  | Anxiety |
|  | Work ability |

## Supplemental Table S14. Weighted ranking of constructs according to REPS, clinical panel members

| **Rank** | **Construct** |
| --- | --- |
| 1 | Work life balance |
| 2 | Job satisfaction |
| 3 | Quality of life |
| 4 | Mental health |
| 5 | Meaning at work |
| 6 | Motivation |
| 7 | Work culture |
| 8 | Income satisfaction |
| 9 | Perceived professional growth |
| 10 | Stress |
| 11 | Participation in decision making |
| 12 | Burnout |
| 13 | Satisfaction with career identity |
| 14 | Sleep |
| 15 | Perceived fulfilment |
| 16 | Work engagement |
| 17 | Job insecurity |
| 18 | Belongingness |
| 19 | Job fit |
| 20 | Perceived support from colleagues |
| 21 | Work related energy |
| 22 | Perceived fairness in workplace |
| 23 | Happiness |
| 24 | Self care |
| 25 | Perceived organizational support |
| 26 | Experienced collaboration with colleagues |
| 27 | Work values |
| 28 | Emotional intelligence |
|  | Interpersonal behaviour |
| 29 | Psychological distress |
| 30 | Fairness of compensation relative to workload |
| 31 | Perceived quality of leadership |
| 32 | Resilience |
|  | Satisfaction with performance at work |
| 33 | Satisfaction with supervisor |
| 34 | Mentorship |
| 35 | Professional identity |
| 36 | Self confidence in professional role |
| 37 | Self efficacy |
|  | Perceived balance between effort and reward |
|  | Fatigue |
|  | Professional values |
|  | Patient-HCP-ratio |
| 38 | Team vitality |
|  | Cognitive overload at work |
| 39 | Climate |
| 40 | Rewarding |
|  | Work excitement |
| 41 | Satisfaction with compensation model |
|  | Professional commitment |
|  | Professional equality |
| 42 | Coping |
|  | Social connectedness at work |
| 43 | Functional status |
|  | Psychological flexibility |
|  | Depression |
|  | Need for recovery |
|  | Previous traumatic work experiences |
| 44 | Work ability |
|  | Anxiety |
|  | Enthusiasm |
|  | Procedural justice |

## Supplemental Table S15. Top 10 constructs per geo-economic stakeholder group (all panel members)

| High geo-economic (n=24) | | | N ranked | % ranked top 10 |
| --- | --- | --- | --- | --- |
|  | 1 | Work life balance | 21 | 87.5 |
|  | 2 | Job satisfaction | 13 | 54.2 |
|  | 3 | Meaning at work | 11 | 45.8 |
|  | 4 | Perceived professional growth | 11 | 45.8 |
|  | 5 | Mental health | 9 | 37.5 |
|  | 6 | Work culture | 9 | 37.5 |
|  | 7 | Motivation | 8 | 33.3 |
|  | 8 | Stress | 8 | 33.3 |
|  | 9 | Income satisfaction | 7 | 29.2 |
|  | 10 | Quality of life | 7 | 29.2 |
| Middle geo-economic (n=9) | | |  |  |
|  | 1 | Work life balance | 7 | 77.8 |
|  | 2 | Income satisfaction | 6 | 66.7 |
|  | 3 | Job satisfaction | 6 | 66.7 |
|  | 4 | Mental health | 6 | 66.7 |
|  | 5 | Motivation | 6 | 66.7 |
|  | 6 | Quality of life | 4 | 44.4 |
|  | 7 | Burnout | 3 | 33.3 |
|  | 8 | Happiness | 3 | 33.3 |
|  | 9 | Meaning at work | 3 | 33.3 |
|  | 10 | Perceived fulfilment | 3 | 33.3 |
| Low geo-economic (n=13) | | |  |  |
|  | 1 | Quality of life | 11 | 84.6 |
|  | 2 | Job satisfaction | 9 | 69.2 |
|  | 3 | Motivation | 7 | 53.8 |
|  | 4 | Work life balance | 7 | 53.8 |
|  | 5 | Income satisfaction | 6 | 46.2 |
|  | 6 | Mental health | 6 | 46.2 |
|  | 7 | Belongingness | 4 | 33.3 |
|  | 8 | Burnout | 4 | 33.3 |
|  | 9 | Fairness of compensation relative to workload | 4 | 33.3 |
|  | 10 | Meaning at work | 4 | 33.3 |

## Supplemental Table S16. Top 10 constructs per geo-economic stakeholder group (clinical panel members)

| High geo-economic (n=18) | | | N ranked | % ranked top 10 |
| --- | --- | --- | --- | --- |
|  | 1 | Work life balance | 17 | 94.4 |
|  | 2 | Job satisfaction | 9 | 50 |
|  | 3 | Meaning at work | 9 | 50 |
|  | 4 | Mental health | 7 | 38.9 |
|  | 5 | Perceived professional growth | 7 | 38.9 |
|  | 6 | Stress | 7 | 38.9 |
|  | 7 | Work culture | 7 | 38.9 |
|  | 8 | Motivation | 6 | 33.3 |
|  | 9 | Quality of life | 6 | 33.3 |
|  | 10 | Experienced collaboration with colleagues | 5 | 27.8 |
| Middle geo-economic (n=7) | | |  |  |
|  | 1 | Job satisfaction | 6 | 85.7 |
|  | 2 | Work life balance | 6 | 85.7 |
|  | 3 | Income satisfaction | 5 | 71.4 |
|  | 4 | Mental health | 4 | 57.1 |
|  | 5 | Motivation | 4 | 57.1 |
|  | 6 | Quality of life | 4 | 57.1 |
|  | 7 | Happiness | 3 | 42.9 |
|  | 8 | Satisfaction with career identity | 3 | 42.9 |
|  | 9 | Burnout | 2 | 28.6 |
|  | 10 | Emotional intelligence | 2 | 28.6 |
| Low geo-economic (n=11) | | |  |  |
|  | 1 | Job satisfaction | 9 | 81.8 |
|  | 2 | Quality of life | 9 | 81.8 |
|  | 3 | Work life balance | 7 | 63.6 |
|  | 4 | Motivation | 6 | 54.5 |
|  | 5 | Mental health | 5 | 45.5 |
|  | 6 | Belongingness | 4 | 36.4 |
|  | 7 | Burnout | 4 | 36.4 |
|  | 8 | Fairness of compensation relative to workload | 4 | 36.4 |
|  | 9 | Income satisfaction | 4 | 36.4 |
|  | 10 | Self care | 4 | 36.4 |

## Supplemental Table S17. Top 10 constructs within professional role

| Allied healthcare professionals/ paramedics (n=6) | | | N ranked | % ranked top 10 |
| --- | --- | --- | --- | --- |
|  | 1 | Job satisfaction | 5 | 83.3 |
|  | 2 | Work life balance | 5 | 83.3 |
|  | 3 | Quality of life | 4 | 66.7 |
|  | 4 | Income satisfaction | 3 | 50.0 |
|  | 5 | Job insecurity | 3 | 50.0 |
|  | 6 | Motivation | 3 | 50.0 |
|  | 7 | Patient-HCP-ratio | 3 | 50.0 |
|  | 8 | Perceived fulfilment | 3 | 50.0 |
|  | 9 | Work related energy | 3 | 50.0 |
|  | 10 | Burnout | 2 | 33.3 |
| Nurses (n=13) | |  |  |  |
|  | 1 | Work life balance | 10 | 76.7 |
|  | 2 | Quality of life | 8 | 61.5 |
|  | 3 | Job satisfaction | 8 | 61.5 |
|  | 4 | Mental health | 8 | 61.5 |
|  | 5 | Motivation | 7 | 53.8 |
|  | 6 | Meaning at work | 5 | 38.5 |
|  | 7 | Work culture | 5 | 38.5 |
|  | 8 | Belongingness | 4 | 30.8 |
|  | 9 | Income satisfaction | 4 | 30.8 |
|  | 10 | Participation in decision making | 4 | 30.8 |
| Physicians (n=14) | | |  |  |
|  | 1 | Work life balance | 13 | 92.9 |
|  | 2 | Job satisfaction | 9 | 64.3 |
|  | 3 | Meaning at work | 7 | 50.0 |
|  | 4 | Income satisfaction | 6 | 42.9 |
|  | 5 | Mental health | 6 | 42.9 |
|  | 6 | Happiness | 5 | 35.7 |
|  | 7 | Perceived professional growth | 5 | 35.7 |
|  | 8 | Work culture | 5 | 35.7 |
|  | 9 | Burnout | 4 | 28.6 |
|  | 10 | Participation in decision making | 4 | 28.6 |
| Researchers (n=10) | |  |  |  |
|  | 1 | Income satisfaction | 6 | 60.0 |
|  | 2 | Mental health | 5 | 50.0 |
|  | 3 | Motivation | 5 | 50.0 |
|  | 4 | Perceived fulfilment | 5 | 50.0 |
|  | 5 | Perceived professional growth | 5 | 50.0 |
|  | 6 | Work life balance | 5 | 50.0 |
|  | 7 | Burnout | 4 | 40.0 |
|  | 8 | Job satisfaction | 4 | 40.0 |
|  | 9 | Meaning at work | 4 | 40.0 |
|  | 10 | Emotional intelligence | 3 | 30.0 |

## Supplemental Table S18. Top 3 ranked constructs within PERMA+4 domains (including all panel members)

| Domain/ constructs | Total points | Total times ranked in top 3 |
| --- | --- | --- |
| **Positive emotions** |  |  |
| 1. Job satisfaction | 96 | 38 |
| 2. Motivation | 57 | 31 |
| 3. Happiness | 39 | 22 |
| **Engagement** |  |  |
| 1. Work life balance | 96 | 36 |
| 2. Perceived quality of leadership | 46 | 25 |
| 3. Participation in decision making | 45 | 23 |
| **Relationship** |  |  |
| 1. Belongingness | 80 | 35 |
| 2. Experienced collaboration with colleagues | 53 | 29 |
| 3. Interpersonal behaviour | 43 | 20 |
| **Mindset** |  |  |
| 1. Mental health | 81 | 29 |
| 2. Resilience | 51 | 23 |
| 3. Self confidence in professional role | 34 | 20 |
| **Accomplishment** |  |  |
| 1. Perceived fulfilment | 61 | 27 |
| 2. Satisfaction with performance at work | 59 | 31 |
| 3. Perceived professional growth | 51 | 24 |
| **Physical health** |  |  |
| 1. Quality of life | 96 | 37 |
| 2. Sleep | 53 | 24 |
| 3. Functional status | 36 | 20 |
| **Economic** |  |  |
| 1. Income satisfaction | 95 | 39 |
| 2. Fairness of compensation relative to workload | 76 | 40 |
| 3. Satisfaction with compensation model | 62 | 32 |
| **Work environment** |  |  |
| 1. Perceived organizational support | 61 | 28 |
| 2. Work culture | 57 | 24 |
| 3. Satisfaction with supervisor | 34 | 16 |

## Supplemental Table S19. Top 3 ranked constructs within PERMA+4 domains (excluding researchers)

| Domain/ constructs | Total points | Total times ranked in top 3 |
| --- | --- | --- |
| **Positive emotions** |  |  |
| 1. Job satisfaction | 77 | 30 |
| 2. Motivation | 48 | 25 |
| 3. Happiness | 29 | 18 |
| **Engagement** |  |  |
| 1. Work life balance | 77 | 28 |
| 2. Perceived support from colleagues | 37 | 21 |
| 3. Participation in decision making | 36 | 18 |
| **Relationship** |  |  |
| 1. Belongingness | 65 | 29 |
| 2. Experienced collaboration with colleagues | 41 | 22 |
| 3. Social connectedness at work | 30 | 14 |
| **Mindset** |  |  |
| 1. Mental health | 64 | 23 |
| 2. Resilience | 35 | 16 |
| 3. Self confidence in professional role | 23 | 14 |
| **Accomplishment** |  |  |
| 1. Satisfaction with performance at work | 46 | 24 |
| 2. Perceived fulfilment | 46 | 20 |
| 3. Perceived professional growth | 37 | 18 |
| **Physical health** |  |  |
| 1. Quality of life | 74 | 29 |
| 2. Sleep | 42 | 18 |
| 3. Work related energy | 31 | 16 |
| **Economic** |  |  |
| 1. Income satisfaction | 71 | 29 |
| 2. Fairness of compensation relative to workload | 59 | 31 |
| 3. Satisfaction with compensation model | 52 | 26 |
| **Work environment** |  |  |
| 1. Perceived organizational support | 53 | 24 |
| 2. Work culture | 48 | 20 |
| 3. Satisfaction with supervisor | 26 | 13 |

## Supplemental Table S20. Top 5 ranked constructs within individual, team or organizational level (all panel members)

|  | Individual | Team | Organizational |
| --- | --- | --- | --- |
| 1 | Job satisfaction (105) | Motivation (66) | Motivation (40) |
| 2 | Work life balance (69) | Job satisfaction (51) | Burnout (39) |
| 3 | Motivation (64) | Perceived support from colleagues (44) | Perceived quality of leadership (38) |
| 4 | Burnout (56) | Participation in decision making (42) | Job insecurity (37) |
| 5 | Happiness (46) | Happiness (40) | Psychological distress (33) |

## Supplemental Table S21. Top 5 ranked constructs within individual, team or organizational level (clinical panel members)

|  | Individual | Team | Organizational |
| --- | --- | --- | --- |
| 1 | Job satisfaction (89) | Motivation (61) | Burnout (32) |
| 2 | Work life balance (58) | Happiness (40) | Motivation (28) |
| 3 | Motivation (52) | Perceived support from colleagues (39) | Perceived quality of leadership (28) |
| 4 | Burnout (39) | Job satisfaction (39) | Work life balance (26) |
| 5 | Happiness (31) | Participation in decision making (38) | Psychological distress (25) |

## Supplemental Table S22. COS-STAR Statement

| Section | Item No. | Checklist item | Application |
| --- | --- | --- | --- |
| **Title/Abstract** |  |  |  |
| Title | 1a | Identify in the title that the paper reports the development of a COS | Page 1 |
| Abstract | 1b | Provide a structured summary | Page 3-4 |
| **Introduction** |  |  |  |
| Background and objectives | 2a | Describe the background and explain the rationale for developing the COS | Page 7-8 |
|  | 2b | Describe the specific objectives with reference to developing a COS | Page 8 |
| Scope | 3a | Describe the health conditions and populations covered by the COS | n/a |
|  | 3b | Describe the interventions covered by the COS | n/a |
|  | 3c | Describe the setting in which the COS is applied | Page 8 |
| **Methods** |  |  |  |
| Protocol/registry Entry | 4 | Indicate where the COS development protocol can be accessed, if available, and the study registration details. | Page 9 |
| Participants | 5 | Describe the rationale for stakeholder groups involved in the COS development process, eligibility criteria for participants from each group, and a description of how the individuals involved were identified. | Page 9 |
| Information sources | 6a | Describe the information sources used to identify an initial list of outcomes. | Page 9 |
|  | 6b | Describe how outcomes were dropped/ combined, with reason (if possible) | Page 9-12 |
| Consensus process | 7 | Describe how the consensus process was undertaken | Page 10-11 |
| Outcome Scoring | 8 | Describe how outcomes were scored and how scores were summarised | Page 10-13 |
| Consensus definition | 9a | Describe the consensus definition | Page 10-11 |
|  | 9b | Describe the procedure for determining how outcomes were included or excluded from consideration during the consensus process. | Page 10-11 |
| Ethics and Consent | 10 | Provide a statement regarding the ethics and consent issue for the study. | Page 9 |
| **Results** |  |  |  |
| Protocol deviations | 11 | Describe any changes form the protocol, with reasons, and describe what impact these changes have on the results. | Page 14 |
| Participants | 12 | Present data on the number and relevant characteristics of the people involved at all stages of COS development. | Page 15-16 |
| Outcomes | 13a | List of all outcomes considered at the start of the consensus process. | Supplementary material |
|  | 13b | Describe any new outcomes introduced and any outcomes dropped, with reasons, during the consensus process. | Page 16-17, and Supplementary material |
| COS | 14 | List the outcomes in the final COS. | Page 17-18 |
| **Discussion** |  |  |  |
| Limitations | 15 | Discuss any limitations in the COS development process. | Page 21-22 |
| Conclusions | 16 | Provide an interpretation of the final COS in the context of other evidence, and implications for future research. | Page 22-23 |
| **Other information** |  |  |  |
| Funding | 17 | Describe sources of funding/ role funders. | Page 23 |
| Conflicts of interest | 18 | Describe any conflicts of interest within the study team and how these were managed. | Page 23 |

## Supplemental Table S23. Final COS items and definitions

| **COS item** | **Short definition** |
| --- | --- |
| Work-life balance | The balance between work demands and personal life responsibilities. |
| Job satisfaction | The degree of contentment employees feel regarding their job. |
| Quality of life | A broad measure of life satisfaction, functioning, and overall health. |
| Motivation | The process that initiates and sustains goal-directed behaviour. |
| Mental health | A state of mind that enables people to cope with stress, function, build relationships, and contribute to their community. |
| Meaning at work | The perceived value, impact, and purpose of one’s work. |
| Income satisfaction | Satisfaction with one’s salary or financial compensation. |
| Work culture | The shared values, norms, and practices within an organization. |
| Perceived professional growth | Perceived progress in professional knowledge, skills, or development. |
| Stress | A mental or emotional response to demanding circumstances. |

# Supplemental Figures

## Supplemental Figure S1. Consensus process for developing the primary COS

## Supplemental Figure S2. Overview of all responses Delphi Round 1

## Supplemental Figure S3. Responses per item from Delphi round 1

1

## Supplemental Figure S4. Overview of all responses Delphi Round 2

## Supplemental Figure S5. Responses per item from Delphi round 2

## Supplemental Figure S6. Top 10 constructs when including all panel members

## Supplemental Figure S7. Top 10 constructs among clinical panel members

## Supplemental Figure S8. Voters by geo-economic group on overall top 10

## Supplemental Figure S9. Voters by geo-economic region on the clinical top 10

## Supplemental Figure S10. Within geo-economic group prioritization of the overall top 10

## Supplemental Figure S11. Within geo-economic group prioritization of the clinical top 10

## Supplemental Figure S12. Within professional-role prioritization of the overall top 10

## Supplemental Figure S13. Within professional-role prioritization of the clinical top 10

## Supplemental Figure S14. Prioritization of constructs across levels of all panel members

## Supplemental Figure S15. Prioritization of constructs across levels excluding researchers

# Appendices

## Appendix A: Additional methodological details

Supplemental Figure 1 gives an overview of the process for developing the COS.

### 1.1 Governance and stakeholder involvement

The steering committee comprised international, multidisciplinary experts in healthcare professional (HCP) wellbeing, health workforce research, public health, and clinical practice. Members included clinicians, researchers, and policy experts from low-, middle-, and high-income settings. The committee defined the scope of the COS, approved the initial construct list and definitions, monitored recruitment and panel composition, and interpreted the consensus results.

Stakeholder panel members were recruited internationally through personal invitations, snowball sampling, and professional networks (including LinkedIn). Eligible participants were physicians, nurses, allied health professionals working in hospital or public health settings, and researchers with expertise in health workforce or wellbeing research. Participants registered via a secure online platform and provided informed consent.

Recruitment aimed to ensure representation across professional roles and geo-economic regions. We targeted at least 30 participants per stakeholder stratum (total ~90–120), in line with recommendations suggesting ~30 panellists per group to support stable consensus within strata.^1,2^ Given our intention to examine both overall consensus and differences across geo-economic settings, this target was not fully achievable across all strata.

**Steering committee members are listed in Appendix C. Panel members who contributed to more than one Delphi round are listed in Appendix D.**

### 1.2 Developing the initial core outcome set

The COS was developed in accordance with COS-STAD recommendations (Supplemental Table S1).

*Identification of outcomes*

Potential wellbeing constructs were first identified through a previously published scoping review by Boskma et al., which systematically mapped instruments used to monitor HCP wellbeing. As this review did not explicitly address economic determinants of wellbeing, an additional focused literature search was conducted to identify constructs related to economic security.

The supplementary search was performed in MEDLINE using terms related to HCP wellbeing and economic determinants (e.g., income, wages, financial status, salaries), aligned with the search strategy and time frame of the original scoping review. The full search strategy is presented in Supplemental Table S2. Articles were eligible if they reported outcomes related to both economic security and HCP wellbeing. Non-English publications were excluded.

The additional search yielded 55 records. After independent title and abstract screening by two reviewers (TB and SM), 16 articles were assessed in full text, of which six met inclusion criteria. From these, six additional economic wellbeing constructs were extracted.

In total, 248 constructs were identified from the original scoping review and supplemented with six economic constructs, resulting in 254 preliminary constructs (Supplemental Table S3).

*Refinement and domain classification*

All identified constructs were reviewed by the steering committee and subsequently independently assessed by three researchers (TB, AB, SM) for relevance to the COS and suitability for Delphi evaluation. During this refinement phase, duplicate, overlapping, or context-specific constructs were removed; closely related constructs were merged; and broadly defined constructs were clarified. Decisions were documented (Supplemental Table S3).

Constructs were provisionally categorized within the PERMA+4 framework (REF BIJ). The domain allocation and definitions were reviewed iteratively with the steering committee, resulting in a final set of 137 unique constructs (Supplemental Table S4), distributed across nine domains: Positive emotions (27), Engagement (12), Relationships (13), Meaning (6), Accomplishment (12), Physical health (13), Mindset (28), Work environment (22), and Economic security (4). A visual overview of the identification and refinement process is presented in Figure 2 of the main manuscript.

### 1.3 Delphi survey design and consensus criteria

All 137 candidate (Supplemental Table S4) constructs were incorporated into a digital survey developed in Castor EDC. Each construct was accompanied by a concise definition and grouped by PERMA+4 domain. Participants were asked to rate the importance of each construct for measuring and describing wellbeing of healthcare professionals in their workplace. This wording was chosen to capture respondents’ own professional judgement rather than perceived external expectations. Each construct was rated on a 9-point Likert scale (1 = not important at all; 9 = very important). A “Don’t know” option was included to avoid forced ratings and reduce artificial central clustering when participants lacked familiarity with a construct.

Consensus thresholds were predefined. Constructs were included, excluded, or carried forward based on percentages of ratings in the 7–9 and 1–3 ranges. In round 2, only constructs meeting inclusion criteria progressed to the final round.

### 1.4 Delphi rounds

*Delphi round 1*

In the first Delphi round, demographic characteristics were recorded and participants were assigned a unique anonymous identifier. All 137 constructs were rated. Participants were also invited to suggest up to four additional constructs they considered important for inclusion.

Round 1 was conducted between July 27 and September 1 2025. Following survey closure, responses were analyzed descriptively and evaluated against the predefined consensus criteria. Constructs meeting inclusion or exclusion thresholds were identified; the remaining constructs progressed to round 2.

Suggestions for new constructs were reviewed and clustered. Constructs suggested by two or more panellists were added to round 2. Two new constructs, “family wellbeing” and “professional equality”, met this criterion and were provisionally categorized within the “Work environment” domain.

*Delphi round 2*

In round 2, conducted between September 25 and October 31 2025, all constructs without consensus were re-evaluated. For each construct, participants were shown the group median score and the distribution of ratings (percentage 1–3 and 7–9). Participants who had completed round 1 also received a summary of their individual prior ratings and were invited to reconsider their responses in light of group feedback.

Newly added constructs from round 1 suggestions were also rated in this round. After closure, data were analyzed descriptively and consensus criteria were reapplied. Only constructs meeting inclusion criteria were carried forward to round 3.

*Delphi round 3: ranking of final COS determination*

After rounds 1 and 2, 62 constructs met inclusion criteria. As a maximum of 10 outcomes had been predefined for the final COS to ensure feasibility and uptake, a ranking procedure was applied in round 3. Round 3 was conducted between November 20, 2025 and January 7 2026.

Panel members were asked to:

1. Select the 10 constructs they considered most important overall;
2. Rank the top three constructs within each PERMA+4 domain;
3. Indicate which constructs were most important at individual, team, and organizational levels.

For the primary determination of the COS, responses from participants who identified their primary professional role as “researcher” were excluded. Although these individuals worked in healthcare-related contexts, the primary objective of the COS was to reflect the perspectives of practicing healthcare professionals. Excluding researchers from the final ranking reduced the risk that results would be driven by theoretical or research-oriented considerations rather than lived professional experience.

To assess the robustness of the prioritization results and explore whether differential representation of stakeholder groups may have influenced rankings, additional analyses were conducted. Weighted ranking analyses in the overall group and the clinicians only group were performed by first calculating, for each construct, the percentage of participants within each geo-economic × professional role subgroup who ranked the construct in their top 10. Subsequently, the mean of these subgroup-specific percentages was calculated per construct to obtain an overall weighted ranking score. In addition to this weighting method, we conducted a weighting using the REPS tool.^3^ This re-weighted priority-setting (REPS) tool, is used in the development of clinical guidelines. In practice, guideline teams use it when they have many candidate items (questions/topics/outcomes) and want a transparent ranking that reduces the chance one stakeholder group dominates the whole list via the tool’s re-weighting mechanism. After each item is selected, the method re-weights participants’ influence based on their support for previously selected ‘winning’ items, helping prevent any single stakeholder group from dominating the final ranking.

References

1	Boulkedid R, Abdoul H, Loustau M, Sibony O, Alberti C. Using and reporting the Delphi method for selecting healthcare quality indicators: a systematic review. *PloS One* 2011; **6**: e20476.

2	Nasa P, Jain R, Juneja D. Delphi methodology in healthcare research: How to decide its appropriateness. *World J Methodol* 2021; **11**: 116–29.

3	Oerbekke MS, Gaasterland CMW, Laan MJ van der, Hooft L. Introducing re-weighted range voting in clinical practice guideline prioritization: Development and testing of the re-weighted priority-setting (REPS) tool. *PLOS ONE* 2024; **19**: e0300619.

## Appendix B: Supplemental results

This section presents detailed Delphi results, subgroup analyses, and supporting tables that complement the summary results reported in the main manuscript.

### 2.1 Participant flow and composition

Participant numbers per round and distribution across professional roles and geo-economic regions are shown in Supplemental Table S5. This overview contextualizes prioritization results by clarifying which stakeholder groups contributed to consensus formation.

### 2.2 Delphi results by round

*Round 1*

Detailed per-item results from round 1, including percentages of ratings in the 1-3 and 7-9 ranges, medians and consensus decisions are presented in Supplemental table S6, and visualized in Supplemental Figure S2 and S3. Free-text suggestions and corresponding core research team decisions are summarized in Supplemental Table S7.

*Round 2*

Item-level results from round 2 are presented in Supplemental Table S8, and visualized in Supplemental Figure S4 and S5.

*Round 3*

Ranking results from round 3 are presented separately for all panel members (Supplemental Table S9) and clinical panel members excluding researchers (Supplemental table S10). Supplemental Figure 6 and 7 present the top 10 constructs for all panel members and for clinical panel members respectively.

In addition, weighted rankings for all panel members and clinical panel members are presented in Supplemental Tables S11 and S12, and in table S13 and S14 for weighting with the REPS tool.

### 2.3 Subgroup and secondary analysis

To explore whether prioritization differed across stakeholder characteristics, additional analyses were conducted.

*By geo-economic region*

Top 10 constructs per geo-economic region among all and clinical panel members is presented in Supplemental table 15 and 16.

Representation of the voters of the overall and clinical top 10 constructs by geo-economic group are presented in Supplemental Figure 8 and 9.

Prioritization within geo-economic regions of the overall and clinical top 10 constructs are presented in supplemental Figures 10 and 11.

*By professional role*

Top 10 constructs per professional role groups are presented in supplemental table 17. Prioritization within professional-roles of the overall and clinical top 10 constructs are presented in Figures 12 and 13.

*Within PERMA+4 domains*

Top 3 constructs per domain are presented for all panel members (Supplemental Table S18) and clinical panel members only (Supplemental Table S19).

*By level of measurement*

Top-ranked constructs at the individual, team, and organizational levels are presented for all panel members (Supplemental Table S20) clinical panel members (Supplemental Table S21) and visualized in Supplemental Figures 14 and 15.

This analysis supports practical implementation by identifying which constructs are perceived as most relevant at different levels of measurement.

## Appendix C. List of Steering committee members

| **Name** | **Country of work** |
| --- | --- |
| Diptesh Aryal | Nepal |
| Amanda Quintairos e Silva | Colombia |
| Jorge Salluh | Brazil |
| Lode Godderis | Belgium |
| Hanan Abdul Rahim | Qatar |
| Meredith Fendt-Newlin | Switserland |
| Yohannes Molla Asemu | Ethiopia |
| Jan Hoving | The Netherlands |
| Rachel Rajadurai | United Kingdom |
| Gotz Wietasch | The Netherlands |

## Appendix D. List of Panel members that contributed to >1 Delphi round

| **Name** | **Country of work** |
| --- | --- |
| Alen Antony Joy | United Kingdom |
| Alexis Germán Murillo Carrasco | Brazil |
| Amanda Drost | the Netherlands |
| Amanuel Sisay Endeshaw | Ethiopia |
| Ana Castillo | Spain |
| Apurb Sharma | Nepal |
| Augusto Ferreira Umpiérrez | Uruguay |
| Bashu Dev Parajuli | Nepal |
| Belete Negese | Ethiopia |
| Beltran Carrillo | Spain |
| Bikila Chemir | Ethiopia |
| Cindy Frias | Spain |
| Cong Wang | Switserland |
| Corey Feist | United States of America |
| Demeke Fentie | Ethiopia |
| Francisco Manuel Olmos Vega | Colombia |
| Getahun Tulu Amante | Ethiopia |
| Getaneh | Australia |
| Habtemaryam Tedla Mekonnen | Ethiopia |
| Hailemariam Getachew Tesema | Sierra Leone |
| Henok Legesse | Ethiopia |
| Ilse Kleibrink | the Netherlands |
| Kalpana Kharbuja | Nepal |
| Kamil Barański | Poland |
| Maharoff Ahameth Nafrin Ali | Sri Lanka |
| Makda Abate Belew | Ethiopia |
| Maria Natividad Seisdedos Núñez | Spain |
| Marieke Dijkman | the Netherlands |
| Marieke Timpener | the Netherlands |
| Maritza Titus | Namibia |
| Matteo Bruschettini | Sweden |
| Meike Slettenhaar | the Netherlands |
| Meseret Yitayew Tizazu | Ethiopia |
| Mulugeta Abate Hailemariyam | Ethiopia |
| Nadine van de Reep | the Netherlands |
| Olivia Lavreysen | the Netherlands |
| Pamela Seron Silva | Chile |
| Peter de Winter | the Netherlands |
| Philip Ayieko | Tanzania |
| Prati Badan Dangol | Nepal |
| Prof. Dr. med. Stefan Schröder | Germany |
| Rana Nashashibi | Palestine |
| Rutger Spruit | the Netherlands |
| Sanne Drost-Goossens | the Netherlands |
| Senta Jorinde Raasveld | the Netherlands |
| Shirish KC | Unites States of America |
| Sithy Zulaiha Zuhair | Sri Lanka |
| Szymon Szemik | Poland |
| Vera Otermann | the Netherlands |
| Yideg Abinew | Ethiopia |

## Appendix E. Decision of medical ethics committee
